# Supplementary material for: Strong electron–phonon coupling in magic-angle twisted bilayer graphene
Source: Nature. 2024 Dec 11;636(8042):342–7. doi: 10.1038/s41586-024-08227-w (PMC11634764; doi:10.1038/s41586-024-08227-w)
Supplement: Supplementary file 1 — Supplementary Information [file 41586_2024_8227_MOESM1_ESM.docx]

**Supplementary Information for**

**Strong Electron-Phonon Coupling in Magic-Angle Twisted Bilayer Graphene**

**Table of contents:**

**I. Absence of superconductivity in the hBN-aligned MATBG**

**II. ARPES spectra analysis**

**III. More ARPES evidence of flat band replicas**

**IV. Evidence of flat band replicas in previous ARPES study**

**V. Possible observation of replica band features in STS**

**VI. Simulated ARPES spectra and its comparison with ARPES result**

**VII. Atomistic modelling of TBG**

**VIII. Moiré electron-phonon model via the frozen phonon assumption**

**IX. Quantum phonon dressing and ARPES spectra in TBG**

**X. Effect of nearly aligned hBN on TBG**

**XI. Impact of twist angle and bandwidth**

**XII Exact diagonalization simulation in the chiral limit**

**XIII Strong correlation between the phonon replicas of the flat bands and superconductivity in MATBG**

**Fig. S1 | STS measurements of MATBG samples.**

**Fig. S2 | Fitting of ARPES spectra.**

**Fig. S3 | ARPES measurement on hBN-unaligned MATBG, Device B.**

**Fig. S4 | Evidence of flat band replicas from data in the literature.**

**Fig. S5 | Possible observation of Replica Features in STS Measurements.**

**Fig. S6| Density dependence of the Amplitude of Peak Features in IETS Spectra.**

**Fig. S7 | Electronic structure of hBN-unaligned and aligned MATBG devices**

**and theoretical simulation.**

**Fig. S8 | Schematic for the reciprocal lattice of TBG.**

**Fig. S9 | Electron phonon coupling in TBG.**

**Fig. S10 | Frozen phonon calculation.**

**Fig. S11 | Determining effective parameters in extended BM model using**

**TAPW method.**

**Fig. S12 | Simulated photoemission spectra.**

**Fig. S13 | Influence of the hBN alignment on the flat band replicas.**

**Fig. S14 | Influence of electronic Coulomb interactions.**

**Fig. S15| A brief summary of current experimental results.**

**Table 1 | Effective parameters extracted from the TAPW method**

**I. Absence of superconductivity in hBN-aligned MATBG**

The alignment of the underlying hBN substrate to MATBG can greatly alter the observed emergent phenomena in MATBG. As revealed in recent transport studies^1,2^, magnetism and an intrinsic quantum anomalous Hall effect are evidenced in MATBG samples aligned to hBN. These observations differ from those typical of unaligned MATBG devices, and likely originate from either the strong influence of the hBN/graphene moiré potential upon the band structure of MATBG, where the two moirés superlattice potentials are commensurate^3,4^, or from the strong influence of the C_2_-symmetry-breaking effects of the atomic-scale stacking configuration of the hBN/graphene moiré, which is expected to change the topological band characteristics of MATBG.

A recent study^5^ using Scanning Tunneling Spectroscopy (STS) has provided a systematic comparison of the electronic structure of hBN-unaligned and hBN-aligned MATBG samples, which are the same samples used in our ARPES study. The key findings are partially reproduced in Fig. S1. In the unaligned sample (Device A&B in our study), the tunnelling gap associated with the superconducting phase is evidenced at filling $-3<v<-2$, consistent with the superconducting density region in the phase diagram of MATBG observed in electrical transport studies^6-8^. This gap feature has been consistently reproduced in several hBN-unaligned MATBG devices^5^, and it is further supported by the presence of a density-tunable Andreev reflection spectrum in point-contact spectroscopy measurements at these fillings. In contrast, the hBN-aligned MATBG (Device C in our work) does not exhibit any evidence of superconductivity or the pseudogap regime within the same density range. This is substantiated by the absence of an Andreev reflection spectrum at these densities^5^. Even the cascade transition of electronic correlated states was not clearly observed in this device, indicating a dramatic change of the electronic structure of MATBG due to the alignment with hBN.


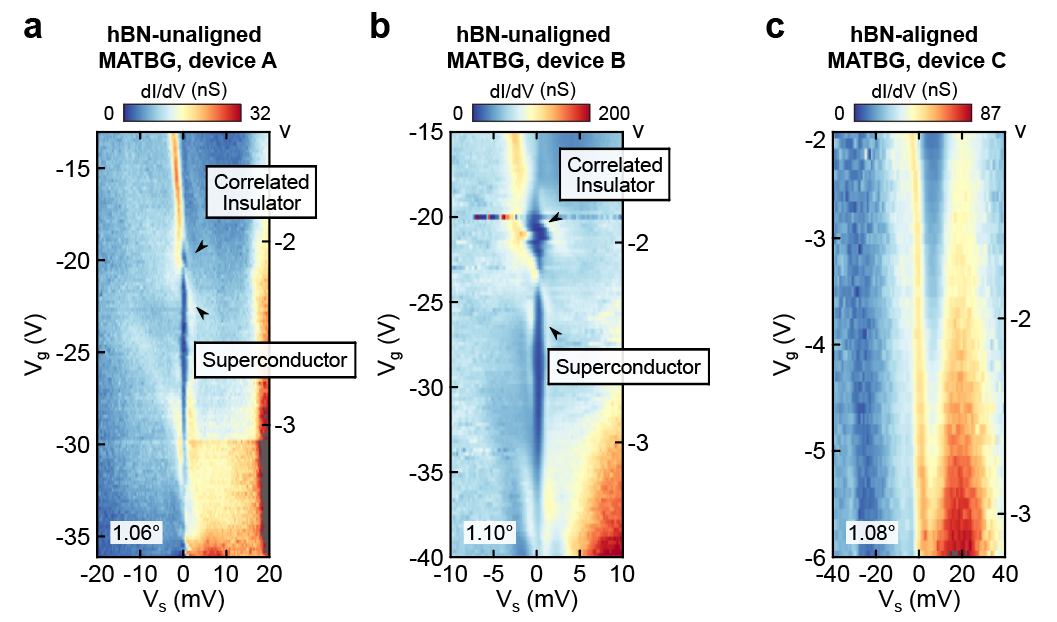


**Fig. S1 | STS measurements of MATBG samples. a**, Tunnelling d*I*/d*V*(*V_s_*, *V_g_*) taken at the center of an AA site in the hBN-unaligned MATBG, Device A (1.06°, 0.1% strain). Arrows label tunnelling gaps observed in the correlated insulating state near v = -2 and in the superconducting state between v = -2 and v = -3, observed at T = 200 mK and B = 0 T. **b**, Tunnelling d*I*/d*V*(*V_s_*, *V_g_*) taken at the center of an AA site in the hBN-unaligned MATBG, Device B (1.10°, 0.1% strain). Arrows label tunnelling gaps observed in the correlated insulating state near v = -2 and in the superconducting state between v = -2 and v = -3, observed at T = 200 mK and B = 0 T. **c**, Tunnelling d*I*/d*V*(*V_s_*, *V_g_*) taken at the center of an AAb site (AA site in the center of a carbon-boron region of the graphene-hBN moiré superlattice) in the hBN-aligned MATBG, Device C (1.08° G–G twist angle, 0.1% G–G interlayer strain, 0.5 ± 0.1° G–hBN twist angle), which used a graphite gate instead of a silicon gate. No gaps were observed in the lower flat band of this device at T = 200 mK and B = 0 T. These figures are reproduced from the main figures (a,c) and supplementary figures (b) of Ref. 5.

**II. ARPES spectra analysis**

To enhance the clarity of the observed flat band replicas, we conducted a paralleled analysis of the ARPES spectra obtained from superconducting MATBG (hBN-unaligned), Device A (Fig. 2S**a-b**) and non-superconducting MATBG (hBN-aligned), Device C (Fig. 2S**c-d**) MATBG. Firstly, we plot the integrated energy distribution curves (EDCs, Fig. S2**b**(i)), obtained from the region denoted by the green dashed line (illustrated in Fig. S2**a** for ‘cut 2’) for all the six dispersion cuts depicted in Fig. 2**c** of the main text. Then, we remove the contribution from the dispersive bands of twisted bilayer graphene, treated as a smooth background, by subtracting a 4^th^-degree polynomial fitting function (Fig. S2**b**(ii)). The background-subtracted EDCs show the intensity of the flat band and its replicas. Importantly, these features appear at the same energy positions throughout the momentum space, exhibiting an energy separation of approximately 150 ± 15 meV, as indicated by the grey dashed lines. This energy separation is further corroborated by the integrated intensity curves of the 2^nd^ derivative of ARPES spectra, as presented in Fig. S2**b**(iii).

In comparison, we applied the same analytical method to the ARPES spectra obtained from the non-superconducting MATBG (hBN-aligned), Device C, and the result is illustrated in Fig. S2**c-d**. In these spectra, other than the original flat band’s peak near *E_F_*, the positions of high binding energy EDC peaks exhibit a pronounced momentum dependence, showing a characteristic behavior of typical band hybridization.


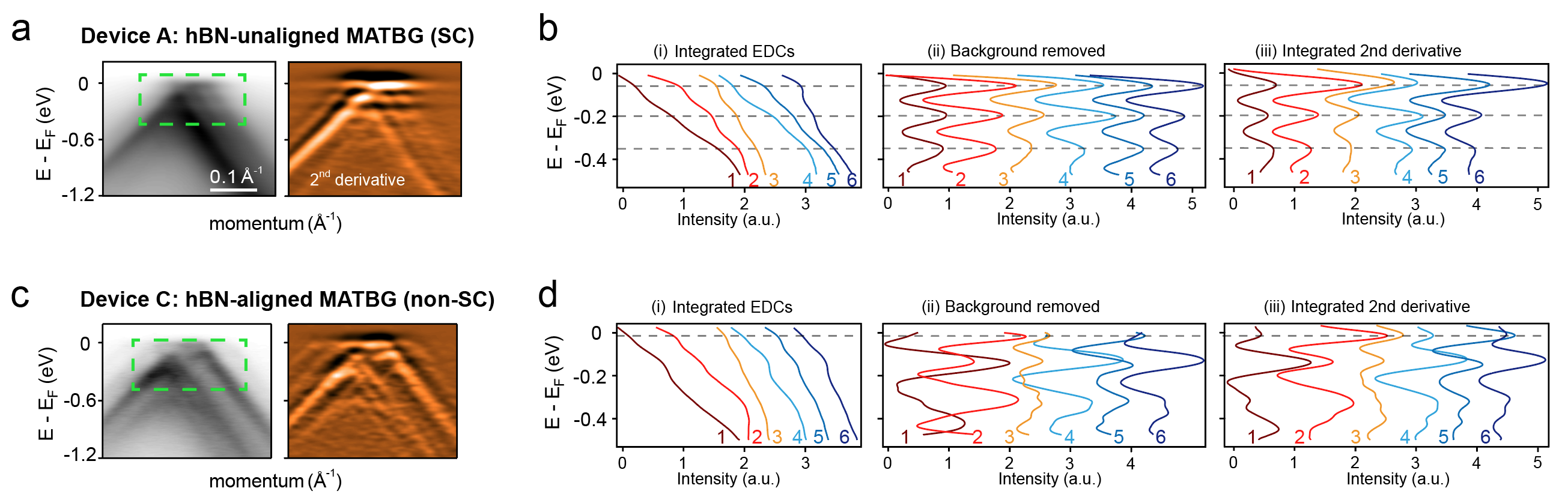


**Fig. S2 | Fitting of ARPES spectra. a-b,** Data analysis on the ARPES spectra taken from superconducting MATBG (hBN-unaligned, Device A) sample. (i) Integrated Energy Distribution Curves (EDCs) for the intensity taken from the green dotted box, for all dispersion cuts in Fig. 2c of the main text (here, only ‘cut 2’ is shown). (ii) the smooth background (4^th^-degree polynomial) removed spectrum of the EDCs in (i), emphasizing the replica bands. Energy separation of approximately 150 meV are indicated by the grey dashed lines. (iii) Integrated intensity of 2^nd^ derivative plots. **c-d**, Same as (a-b) but for ARPES spectra taken from non-superconducting MATBG (hBN-aligned, Device C) sample.

**III. More ARPES evidence of flat band replicas**

Figure S3 presents detailed result of ARPES measurement conducted on Device B, a supplementary to the Fig. 2e of the main text. This device is a superconducting MATBG sample (hBN-unaligned), where the twist angle is determined to be 1.10° within the magic region and superconducting state has been confirmed by the STS result, detailed in Fig. S1. Optical image and corresponding ARPES real space spectra map are shown in Fig. S3**a**. The ARPES dispersion plots that showing the observation of flat band and its replicas are presented in the Fig. 2**e** of the main text. Here, we perform similar data analysis to extract the energy positions of the flat band replicas as in previous sections (SI-Section II). Since the Fermi energy of this particular device intersects the flat bands, we divide the energy resolution convolved Fermi-Dirac distribution to restore the ARPES intensity around and above the Fermi energy^9^. The results, shown in Fig. S3**c**, display an energy separation for the flat band and its replicas of approximately 150 meV, which is consistent with the findings in Device A, further substantiating the presence of flat band replicas in MATBG.

Since the *E_F_* of MATBG/hBN devices resides at the bottom of the flat band – as ARPES technique can only probe the occupied electronic states – in order to observe the flat band spectra (thus also its replicas) we need to elevate the measurement temperature to thermally populate valance electrons to the flat band. This approach allows us to unveil the electronic structure slightly above *E_F_* (within the range of a few *k_B_T*). Indeed, measuring at higher temperatures will slightly worsen the energy resolution, but thanks to the relatively large separation between the replicas (the phonon mode energy of ~150 meV), we were still able to observe the flat bands with replicas, showing the robustness of the EPC in this system.

In Fig. S3e, we present the results of temperature-dependent measurements conducted on Device B. When the temperature is lowered to 20K, the flat band is no longer observed due to the cutoff associated with the Fermi energy (the thermal excitation is insufficient at this reduced temperature). Importantly, the flat band replicas are also absent at this lower temperature, and their behavior is correlated with the absence of the main flat band. This observation further supports the hypothesis that these replica features are associated with the flat band in MATBG.


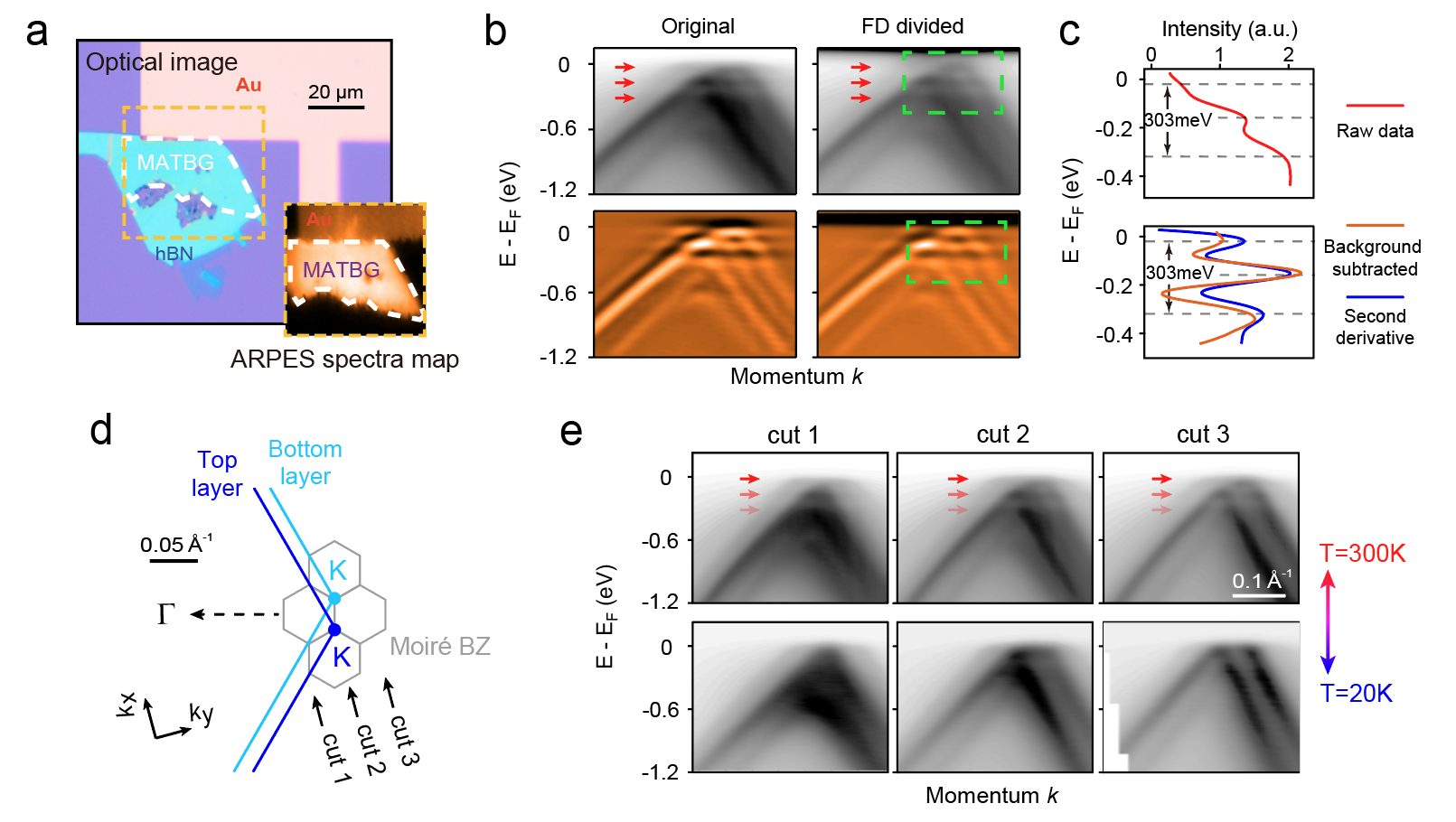


**Fig. S3 | ARPES measurement on hBN-unaligned MATBG, Device B. a,** Optical image and corresponding ARPES spectra map of the MATBG device. **b**, Left column: ARPES spectra plot and 2^nd^ derivative plot (cut 2, illustrated in Fig. 2e of main text). Right column: corresponding ARPES and 2^nd^ derivative plot divided by the energy resolution convolved Fermi-Dirac distribution. The high statistic of the measurement at room temperature enables the spectra restoration above Fermi energy, due to thermal excitation. **c,** Top panel: Integrated ARPES spectra intensity in the green dashed line box in (b). Bottom panel: The red curve represents the same data shown in the top panel with the removal of a smooth background (4^th^-degree polynomial). The blue curve represents the integrated intensity of the 2^nd^ derivative plot within the same green dashed line box. **d,** Illustration of the MATBG moiré BZs around the K point of top and bottom monolayer graphene. Grey lines show the moiré BZs of MATBG. ‘cut1’ ~ ‘cut3’ mark the coordinates of ARPES spectra in (e). **e,** Temperature-dependent ARPES spectra (momentum directions indicated in Fig. 2 of the main text) taken at 300 K and 20 K, respectively. The replicas show correlated behavior with the main flat bands, absent at low temperatures.

**IV. Evidence of flat band replicas in previous ARPES study**

In Fig. S4, we present an analysis of ARPES data (with the authors’ consent) from a previous study^10^, focusing on the electronic structure of MATBG. This particular MATBG device, with a twist angle of approximately 0.96° (± 0.03°) within the magic region, is unaligned with the hBN substrate (with a twist angle of 3°), and therefore expected to exhibit a superconducting state^5^ (although not characterized in their study). The figure shows ARPES spectra taken at consecutive parallel momenta in the vicinity of the graphene K point. The flat band around the Fermi energy and the first-order phonon replicas (indicated by the arrows) are evidenced, which is more prominent in the 2^nd^ derivative plots. The energy separation between these features is approximately 160 meV, consistent with the results obtained from our ARPES data. Notably, in this sample, the second-order replica is relatively weak and is not as visible as in our result from Device A (1.08°) and Device B (1.10°). This is consistent with its smaller twist angle (0.96°) which slightly deviates from the optimal value of 1.10°. The impact of twisted angle/bandwidth on the EPC strength will be discussed in detail in Sec. XI.


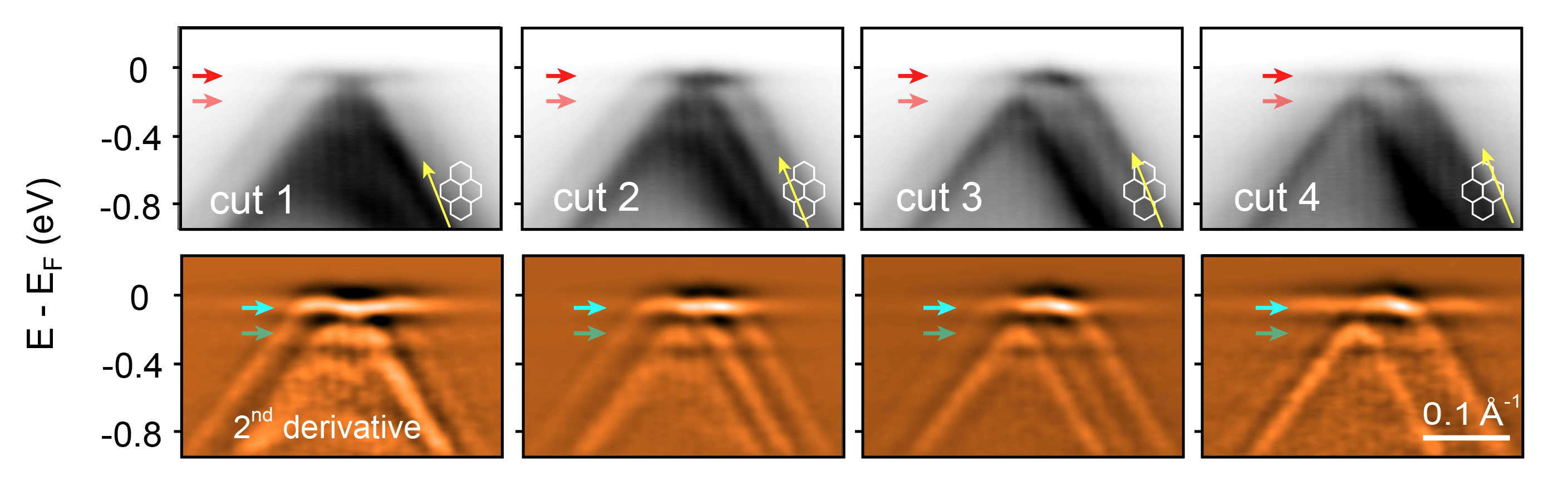


**Fig. S4 | Evidence of flat band replicas from data in the literature.** ARPES spectra plots and 2^nd^ derivative plots along consecutive parallel momentum directions. The flat band and its first-order phonon replica are evidenced. The original data is taken from a previous work^10^ (with the authors’ consent), on a MATBG sample (0.96 ± 0.03°) with unaligned hBN substrate (3°).

**V. Possible observation of replica band features in STS**

In a continued effort to explore the presence of phonon-replica bands, as observed in ARPES measurements on superconducting MATBG devices, we conducted STS measurements on a superconducting MATBG device (hBN-unaligned, Device F). While STS is not inherently sensitive to the electronic structure around the graphene K point, it still offers valuable insights.

As depicted in Fig. S5, the STS measurements revealed multiple peaks that are evenly spaced in energy, with an approximate separation of 150 meV, which could be related to the observations of replica bands in ARPES measurements. Importantly, these features exhibit a strongly correlated behaviour with the main flat band, which suggests that they are related to phonon replicas instead of band hybridization effects. However, other possible mechanisms could also account for these STS peaks which are more difficult to disentangle by STM measurements alone, as discussed below.

In Fig. S5**a**, we show the experimental setup for this measurement. STS measurements are typically performed using a standard lock-in measurement scheme, where an AC modulation voltage V_rms_ at frequency ω is added to the DC sample voltage V_s_, resulting in an AC tunneling current between the tip and sample. Demodulating this current signal at the same frequency ω measures the differential conductance dI/dV(V_s_, V_g_), which yields information about the local density of states (in the single-particle limit). Demodulating at the higher-order frequency 2ω measures the inelastic tunneling spectrum (IETS) d^2^I/dV^2^(V_s_, V_g_), which can yield information about inelastic tunneling processes or dressed electronic states.

In Fig. S5**b**, we show a dI/dV(V_s_, V_g_ = 0 V) spectrum obtained near charge neutrality in MATBG, which shows a total of seven dominant peaks (six at negative V_s_ and one at positive V_s_). The two peaks closest to E_F_ are typical of STS measurements on graphene, and have been attributed to inelastic tunneling processes mediated by acoustic graphene phonon modes near either the K- or M-point of the graphene Brillouin zone^11^. These modes are not particular to measurements on MATBG, and are distinct from the intervalley K-point phonon mode reported in the main text. The remaining peaks (five at higher negative sample voltages) are not observed in monolayer graphene. IETS measurements in this device location at the same gate voltage (Fig. S5**c**) reveal IETS dips evenly separated by roughly 150 meV in energy. In measuring the density dependence of these features in both dI/dV(V_s_, V_g_) (Fig. S5**d**, left) and in d^2^I/dV^2^(V_s_, V_g_) (Fig. S5**d**, middle and right), we find the spectral intensity of these features to parallel the spectral intensity of the flat bands of MATBG near the Fermi level. Particularly, as the flat bands of MATBG shift in energy across the Fermi level, the intensity of each IETS feature below E_F_ decreases. This provides further evidence that these peaks likely do not represent features of the local density of states (ex. band hybridization effects), and are suggestive of replica features related to the flat bands’ states themselves.

In order to extract the energy positions and spacing of IETS peaks from d^2^I/dV^2^(V_s_, V_g_) measurements, we perform a multi-peak Voigt fitting procedure, as detailed below. At each gate voltage, we first identify the sample voltages of local minima in d^2^I/dV^2^(V_s_) spectra. We use these local minima locations to seed a model function (“VoigtModel” imported from the “lmfit” Python package for non-linear least-squares minimization), which identifies the peak energy positions of IETS features as a function of gate voltage. In the right panel of Fig. S5**d**, we plot the energies of IETS peaks at each gate voltage (black dot markers), as extracted from the multi-peak Voigt fitting procedure. As the gate voltage changes the system’s chemical potential, the flat bands and the IETS peak features shift almost rigidly in energy. By subtracting the energies of the IETS features closest to E_F_ (also extracted using the same Voigt fitting procedure) from the energy of the higher-energy IETS feature appearing at the same density, we remove most of this chemical potential dependence of the energy of each peak in IETS to provide an estimate of the density-independent energy spacing of 150 ± 5 meV of the features in d^2^I/dV^2^(V_s_, V_g_) measurements.

Besides their plausible attribution to the replica flat bands observed in ARPES, we have considered alternative mechanisms that could be responsible for these peaks in STS and IETS measurements. Particularly, peaks in STS that are evenly spaced in energy could plausibly be attributable to so-called “whispering gallery modes” (WGMs) previously seen in tip-induced or electrostatically defined PN junctions in graphene devices^12-15^. The following discussion highlights several key features of our density-dependent spectroscopic measurements (Fig. S5d) that should be considered when comparing these competing hypotheses.

First, at each gate voltage, the IETS peak features we observed in MATBG are asymmetric with respect to the sample’s bias voltage polarity, observed only at negative sample bias voltages (Fig. S5b,c). WGM resonances are typically reported to cluster near V_s_ = 0 V, and are observed to be evenly spaced in energy both above and below V_s_ = 0 V (see Fig. 2a,b of Ref. 12; Fig. 4a of Ref. 13; Fig. 3g of Ref. 14; Fig. 1f of Ref. 15). However, this is not a necessary condition for WGMs, which can appear asymmetric with respect to the bias voltage for particular configurations of tip-sample junction potentials.

Second, in addition to their energy spacing being density-independent, the IETS peak features occur at energies that are also nearly density independent over a wide range of gate voltages (right panel of Fig. S5d); whereas, previous observations of WGMs have shown them to rapidly disperse in energy as a function of density while maintaining their constant energy spacing from each other (ex. Fig. 2a,b of Ref. 12).

Third, we have taken careful measures to work function match our STM tips with the graphene sample in order to avoid spurious effects from local STM tip gating. In each measurement location, we have verified the negligible influence of the STM tip on the sample after work function matching via the linearity of the filling of MATBG with applied gate voltage (see Extended Data Fig. 6 in Ref. 16 for examples of nonlinear gating and tip-induced band bending in MATBG).

Finally, as a function of density (Fig. S5d), we observed a correspondence between the amplitudes of the IETS peak features (five large triangle markers; Fig. S6) and that of the typical phonon-assisted tunneling peak observed in all graphene samples at ±60 meV with respect to the energy of any elastic tunneling channel (small triangle marker; Fig. S6). Both features decrease in amplitude with decreasing gate voltage as the flat bands of MATBG are gated across the Fermi level. This behavior suggests that all of these IETS features are connected to the density of states of MATBG’s flat bands. Note that the appearance of the replica features in ARPES measurements rules out the possibility of these features being similarly attributed to phonon-assisted tunneling processes.

Although these STS results parallel observations in ARPES measurements, considering the complexity involved in interpreting the data, including the influence of other acoustic phonons on the visualization of the electronic structure at the K point of graphene^11^, as well as to fully understand the role of various experimental factors (ex. device twist angle or strain, STM tip condition), it is clear that additional STS investigations are warranted to gain deeper insights of the observed IETS features.


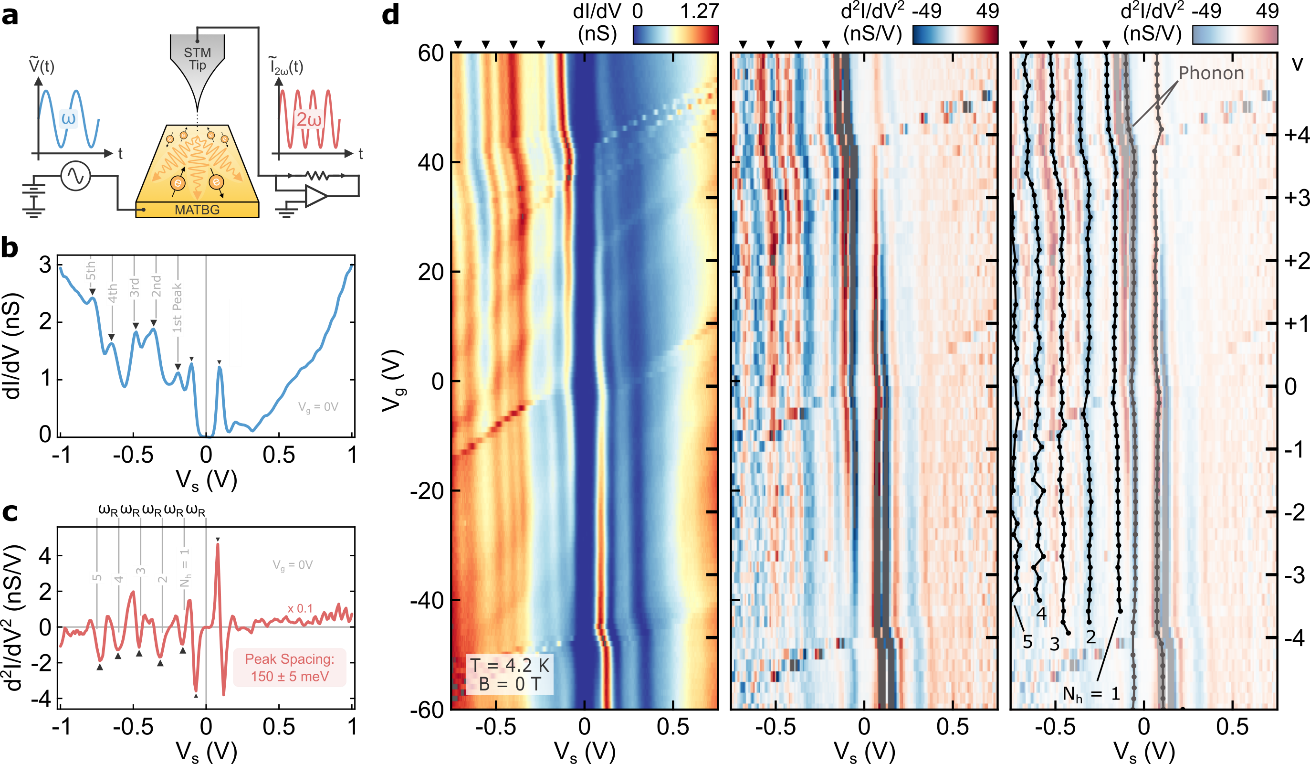


**Fig. S5 | Possible observation of Replica Features in STS Measurements. a,** Schematic of the experimental setup. Inelastic tunneling spectroscopy (IETS) measurements were performed using a lock-in technique where an AC voltage at frequency ω is applied to the sample, and the resulting AC tunneling current between the tip and sample is demodulated at frequency 2ω. **b,** dI/dV(V_s_, V_g_ = 0 V) obtained at the center of an AA site over a wide voltage bias range. The two IETS features that flank V_s_ = 0 V (small triangle markers) are typical of STS measurements on graphene, and are related to inelastic tunneling due to K- or M-point phonons. The five additional features below E_F_ are particular to MATBG, and are separated in energy by roughly 150 meV. **c,** d^2^I/dV^2^(V_s_, V_g_ = 0 V) obtained at the center of an AA site using the previously described 2^nd^-harmonic lock-in technique. Sharp dips below E_F_ identify peak features in these measurements. **d,** dI/dV(V_s_, V_g_) obtained at the center of an AA site (left panel), which depicts the density dependence of the observed replica band features. d^2^I/dV^2^(V_s_, V_g_) obtained at the center of an AA site (raw data in the middle panel; annotated data in the right panel), which shows similar density dependence as observed in dI/dV(V_s_, V_g_). IETS dip features are annotated in each spectrum in the right panel using extracted peak locations from a multi-Voigt fitting procedure.


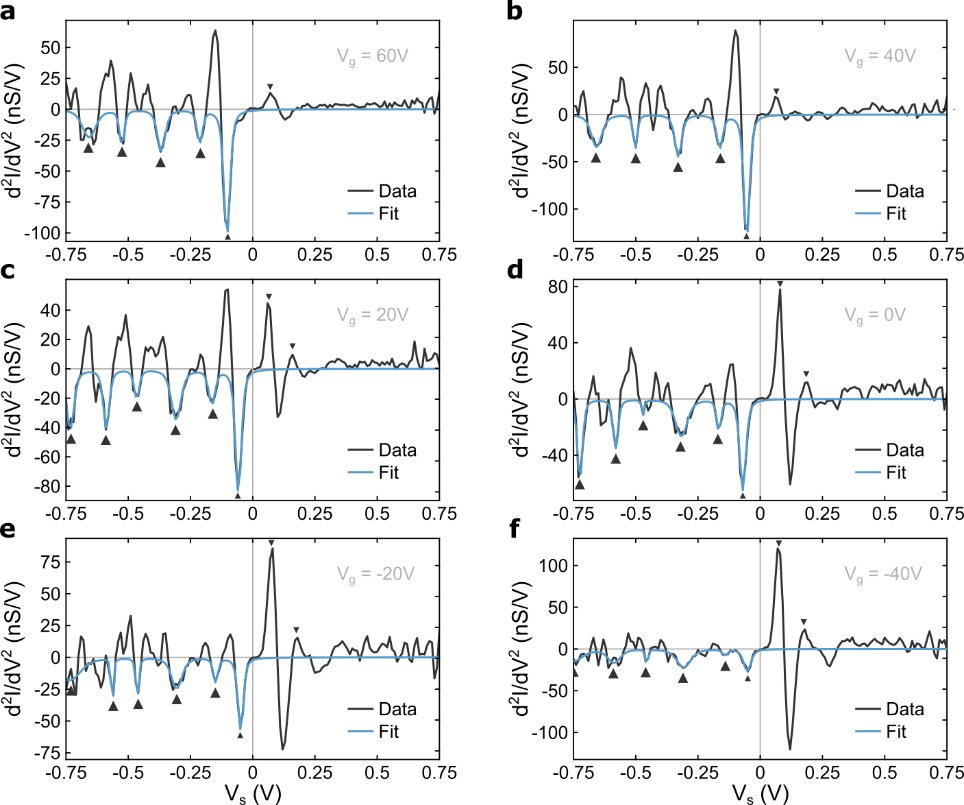


**Fig. S6| Density dependence of the Amplitude of Peak Features in IETS Spectra.** Raw IETS spectra d^2^I/dV^2^(V_s_, V_g_) (black) and multi-peak Voigt fits (blue) taken from Fig. S5d at V_g_ = 60 V (a), 40 V (b), 20 V (c), 0 V (d), -20 V (e), and -40 V (f). Large triangle markers identify higher-energy IETS peak features and small triangle markers identify phonon-assisted tunneling features. As gate voltage decreases, all IETS dips observed at V_s_ < 0 V decrease in amplitude while shifting minimally in energy over a wide range of densities.

**VI. Simulated ARPES spectra and its comparison with ARPES result**

We have simulated the basic electronic structure of both hBN-aligned (0.5° twist) and hBN-unaligned (8° twist) MATBG based on the continuum model (presented in Fig. S7), without considering any correlation effects. The simulation, on the single-particle level, generally captures the overall electronic structure of the MATBG system, including the hybridized Dirac-shape bands and their moiré replicas in momentum space.

To compare with the experiments, in Fig. S7a we show three simulated dispersions cutting along the same direction and places in the momentum space as in the experiments (indicated in Fig. S7b, same as main text Fig. 2 and Fig. 3), which were then broadened to the resolution comparable to the experiments (see Fig. S7c). Clearly, even in the broadened spectra in Fig. S7e, there are no spectra features/signatures like the flat band replicas observed in the experiment (see Fig. S7d). This indicates that the observed flat band replicas originate from strongly correlated effect beyond typical band hybridizations.

Besides, the simulation also shows that the alignment of the hBN-substrate has substantial effects in the band structure of MATBG. As can be seen in Fig. S7a, the simulated band dispersions of the isolated MATBG (top row) and hBN-unaligned MATBG (middle row) are very similar, for both the flat band and the complicated valance band. However, when the hBN-substrate is aligned with the MATBG, the energy gap between the flat band increase (Fig. S7a, in particular the zoomed in plots on the right column), further separating the upper and lower flat band around Fermi level (E_F_). This difference is due to the additional moiré potential between the aligned hBN-substrate and graphene, whose length scale is almost identical to that of the MATBG moiré superlattice.

Apart from the effect on the flat bands, the aligned hBN/MATBG moiré potential can further affect the valance bands, resulting in some redistributions of spectral intensities (Fig. S7a and c). Remarkably, the influence of the enhanced moiré potential due to the aligned-hBN substate can also be seen in the spectral intensity span over the moiré BZs: as illustrated in Fig. S7e and f, both the simulation and the experimental results show that the spectral intensity of the hBN-aligned MATBG system span over more moiré BZs.

The results in Fig. S7 clearly demonstrates that the hBN-substrate does have appreciable influence on the MATBG above it, which could help explain the different properties of the hBN-unaligned (superconducting) and hBN-aligned (demonstrating quantum anomalous Hall) MATBG devices.

It is also worth noting that the relatively low resolution of our ARPES experiment, especially the devices-variation, mainly comes from the surface quality of each individual device. Unlike measuring conventional single crystals for which we can in-situ cleave the samples in the measurement chamber to obtain an ultraclean surface, the TBG devices were prepared ex-situ (e.g. in air and/or in the glove box) with multiple steps. Although we can apply complementary surface cleaning techniques, including ex-situ AFM cleaning, in-situ high-temperature annealing, etc., the surface quality of these devices is intrinsically not as good as in-situ cleaved single crystal samples, and can vary between devices.


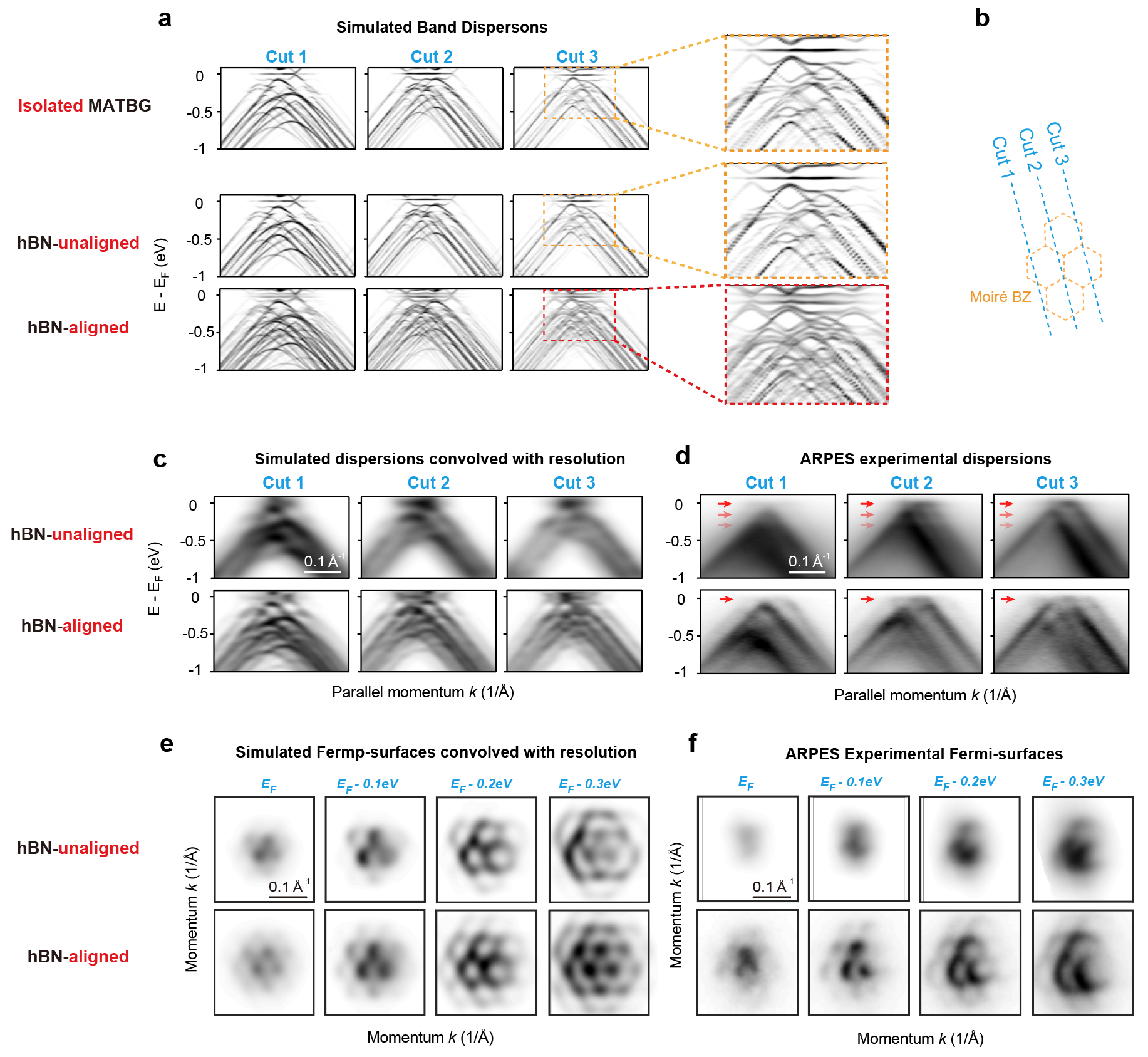


**Fig. S7 | Electronic structure of hBN-unaligned and aligned MATBG devices and theoretical simulation. a,** Simulated band dispersion cuts for isolated, hBN-unliagned, and hBN-aligned MATBG, respectively. **b,** Momentum directions for the cuts illustrated in (a), (e), and (f) (same as main text Fig. 2 and Fig. 3). **c,** Simulated ARPES band dispersion cuts in (a) convolved with Gaussian resolution function (45 meV in energy and 0.02 Å^-1^ in momentum). **d,** Corresponding experimental results for the dispersion cuts (Device A and C, replotted from main text Fig. 2 and 3). **e,** Simulated ARPES constant energy contours (Fermi level (*E_F_*) and 0.1eV, 0.2eV, 0.3eV below) in the vicinity of graphene K point for both hBN-unaligned and aligned MATBG. Gaussian resolution function is convolved. **f,** Corresponding experimental results for the constant energy contours (Device A and C).

**VII. Atomistic modelling of TBG**

The unit lattice vector for the moiré supercell of TBG with twist angle $\theta$ is defined as $\boldsymbol{L}_{\boldsymbol{1}}=L_{\theta}\left( \frac{1}{2},\frac{\sqrt{3}}{2} \right)$, and $\boldsymbol{L}_{\boldsymbol{1}}=L_{\theta}\left( -\frac{1}{2},\frac{\sqrt{3}}{2} \right)$. The moiré lattice constant is $L_{\theta}=a\sqrt{3N^{2}+3N+1}$, with $a=2.46Å$, the monolayer-graphene lattice constant. The corresponding unit reciprocal lattice vectors are

$\boldsymbol{G}_{\boldsymbol{1}}=\sqrt{3}k_{\theta}\left( \frac{\sqrt{3}}{2},\frac{1}{2} \right)$, $\boldsymbol{G}_{\boldsymbol{2}}=\sqrt{3}k_{\theta}\left( -\frac{\sqrt{3}}{2},\frac{1}{2} \right)$, (1)

where $k_{\theta}=4\pi/(3L_{\theta})$. For modeling MATBG, we set $N=30$, i.e. $\theta=1.08^{\circ}$ to calculate the band structure. When the twist angle is small, atomistic fluctuations in the *z* direction may be evident. This corrugation effect can be described by the *z*-direction displacements for carbon atoms^17^

$$d^{(1)}=\frac{1}{2}d_{0}+d_{1}\sum_{n=1,2,3} cos(G_{n}\cdot\tau)$$

$d^{(2)}=-\frac{1}{2}d_{0}-d_{1}\sum_{n=1,2,3} cos(G_{n}\cdot\tau)$. *(2)*

In these expressions, the superscript is the layer index, $\boldsymbol{G}_{\boldsymbol{3}}\boldsymbol{=-(}\boldsymbol{G}_{\boldsymbol{1}}\boldsymbol{+}\boldsymbol{G}_{\boldsymbol{2}}\boldsymbol{)}$ is the third smallest moiré reciprocal lattice vector, and $\tau$ is the atomic position of the carbon atom in the moiré unit cell. Between the two distance parameters, $d_{0}=3.43Å$ is the average interlayer distance for TBG, $d_{1}=0.278Å$ sets the difference of the interlayer distance between AA–stacking and AB–stacking regimes.

Based on the moiré supercell geometry, we can write down the non-interacting Hamiltonian of TBG under tight-binding (TB) approximation

$H= -\sum_{Ii\alpha,Jj\beta} t\left( \boldsymbol{R}_{Ii\alpha}-\boldsymbol{R}_{Jj\beta} \right)c_{Ii\alpha}^{\dagger}c_{Jj\beta}$ *(3)*

where $c_{Ii\alpha}^{\dagger}$, $c_{Jj\beta}$ are creation and annihilation operators for the $p_{z}$ orbital of $i\alpha$ carbon atom in the I-th moiré superlattice ($\alpha$, $\beta$ are joint indices for A/B sublattices and (1)/(2) layers, while $i,j$ label atomic coordinates inside a moiré superlattice. The hopping parameter $t$ is related to the atomic structure through the Slater-Koster formula

$t\left( r \right)= -V_{\pi}\left( 1- \frac{r_{z}^{2}}{r^{2}} \right)-V_{\sigma}\frac{r_{z}^{2}}{r^{2}}$ *(4)*

where $\boldsymbol{r}_{\boldsymbol{z}}=\boldsymbol{r}\cdot\boldsymbol{e}_{\boldsymbol{z}}$, $V_{\pi}=V_{\pi}^{0}e^{-\left( r-a_{0} \right){/r}_{0}}, V_{\sigma}=V_{\sigma}^{0}e^{-\left( r-d_{0} \right){/r}_{0}}$. Specifically, for the TBG, $a_{0}=a/\sqrt{3}$ is the nearest-neighbour distance, $r_{0}=0.184a$is the decay length of the hopping strength; the hopping amplitudes are set as $V_{\pi}^{0}=-2.7\mathrm{eV},V_{\sigma}^{0}=0.48 \mathrm{eV}$^18^. With translational symmetry, the Hamiltonian can be decomposed into momentum-space quadrature $H= \sum_{\bar{\boldsymbol{k}}} c_{\bar{\boldsymbol{k}}}^{\dagger}H_{\bar{\boldsymbol{k}}}^{\left( \mathrm{TB} \right)}c_{\bar{\boldsymbol{k}}}$ for moiré superlattice, with the vector $c_{\bar{\boldsymbol{k}}}$ annihilating an electron at momentum $\bar{\boldsymbol{k}}$ of the mini-Brillouin zone (mini-BZ) and its vector index going through both *i* and *α*. The explicit relation between the TB Hamiltonian and atomic coordinates allows an efficient estimation of the electron-phonon coupling (EPC) in a frozen phonon manner.

We further construct the low-energy effective Hamiltonian similar to the Bistritzer-MacDonald (BM) model using Truncated Atomic Plane Wave (TAPW) method^19^. By restricting the wavefunctions to a series of TAPWs centered at atomic $K$ and $K'$ points, i.e. $c_{\bar{\boldsymbol{k}}}^{\left( \mathrm{TAPW} \right)}=X^{\dagger}c_{\bar{\boldsymbol{k}}}$, the full Hamiltonian Eq. (3) can be projected onto a lower-dimensional effective Hamiltonian

$H_{\bar{\boldsymbol{k}}}^{\left( \mathrm{TAPW} \right)}=X^{\dagger}H_{\bar{\boldsymbol{k}}}^{\left( \mathrm{TB} \right)}X$ , *(5)*

which gives a good approximation for the eigenstates near the Fermi level. Here, $c_{\bar{\boldsymbol{k}}}^{\left( \mathrm{TAPW} \right)}$ and $H_{\bar{\boldsymbol{k}}}^{\left( \mathrm{TAPW} \right)}$ are the annihilation operator and the TB matrix in the TAPW basis, and $X$ is the plane-wave projector defined as ${{(X}_{\alpha})}_{n,i}=e^{i\boldsymbol{G}_{\boldsymbol{n}}\boldsymbol{\tau}_{\boldsymbol{i\alpha}}}/\sqrt{N_{\alpha}}$, where $\boldsymbol{\tau}_{\boldsymbol{i\alpha}}$ denotes the displacement of the α-th atom in the *i*-th unit cell and $N_{\alpha}$ denotes a quarter of the size of a moiré supercell. The reciprocal vector $\boldsymbol{G}_{\boldsymbol{n}}$, as shown in Fig. S8, is centered around atomic $K$ and $K'$ points, and is a linear combination of moiré reciprocal lattice vectors $\boldsymbol{G}_{\boldsymbol{1}}$ and $\boldsymbol{G}_{\boldsymbol{2}}$.


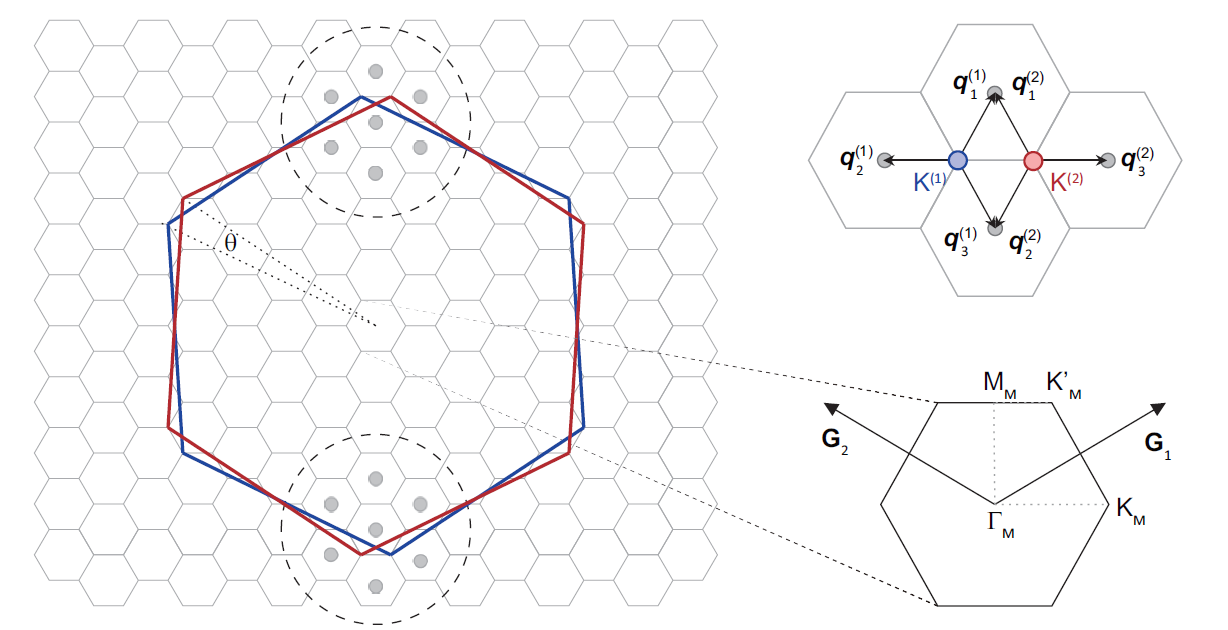


**Fig. S8 | Schematic for the reciprocal lattice of TBG**. The twisted angle is set to be $\theta=7.341^{\circ}$ for better illustration. The projection is constructed using a series of TAPWs in two circled areas for different atomic valleys $K$ and $K'$. The large and small hexagons represent the reciprocal lattice for (twisted) monolayer graphene and moiré superlattice. High symmetry points of the moiré reciprocal lattice are denoted as Γ_M_, M_M_, K_M_, and K’_M_. $\boldsymbol{G}_{\boldsymbol{1}}\boldsymbol{,}\boldsymbol{G}_{\boldsymbol{2}}$ are moiré reciprocal lattice vectors. The small $q$ vectors, which connect a K point to their nearest Γ points and modulate the moiré periodicity for the phonons, are also presented.

**VIII. Moiré electron-phonon model via the frozen phonon assumption**

Previous studies^20-22^ based on large-scale molecule dynamics (MD) simulations point out that the density of states of MATBG at high frequency is almost the same as bilayer graphene, suggesting that the impact of moiré potential on high-frequency phonons is negligible. Then, we can mimic phonons at the moiré Γ_M_ point, which results from iTO/iLA/iLO phonons at graphene’s $K$ and $K'$ points through a folding. The real-space atomic displacement $\eta\left( r \right)$ induced by the moiré phonons at the Γ_M_ point can be generated in the following way:

$\eta^{(l)}\left( r \right)=s\left[ \left( e^{i\boldsymbol{q}_{1}^{\left( l \right)}\cdot\boldsymbol{r}}+e^{i\boldsymbol{q}_{2}^{\left( l \right)}\cdot\boldsymbol{r}}+e^{i\boldsymbol{q}_{3}^{\left( l \right)}\cdot\boldsymbol{r}} \right)e^{i\boldsymbol{K}^{\left( l \right)} \cdot\boldsymbol{r}} u_{\alpha}+c.c. \right]\delta(\boldsymbol{r}-\boldsymbol{r}_{i\alpha})$ *(6)*

where $u_{\alpha}$ is the polarization vector for $\alpha$-th carbon atom of the monolayer graphene at momentum $K$ or $K'$ point; $e^{i\boldsymbol{q}\cdot\boldsymbol{r}}$ is the moiré modulation factor, and $s$ is the distortion strength. The EPC strengths can be estimated by feeding updated atomic positions to TAPW Hamiltonian matrix $H_{\bar{\boldsymbol{k}}}^{\left( \mathrm{TAPW} \right)}$ after considering in-plane lattice distortion, denoted as $H_{\bar{\boldsymbol{k}}}^{\mathrm{eph}}$. By comparing the relative change of phonon-influenced electronic Hamiltonian matrix (at K_M_ and Γ_M_ points), ${||H_{\bar{\boldsymbol{k}}}^{\mathrm{eph}}-H_{\bar{\boldsymbol{k}}}^{\left( \mathrm{TAPW} \right)}||}_{2}/{||H_{\bar{\boldsymbol{k}}}^{\left( \mathrm{TAPW} \right)}||}_{2}$ (where ${||\cdot||}_{2}$ represents for *L_2_* norm of the matrix), we find the flat-band electrons have much stronger coupling (reflected by the slop of energy over displacement) with iTO moiré phonons than other ones (iLO/iLA), as shown in panel (b) of Fig. S9.


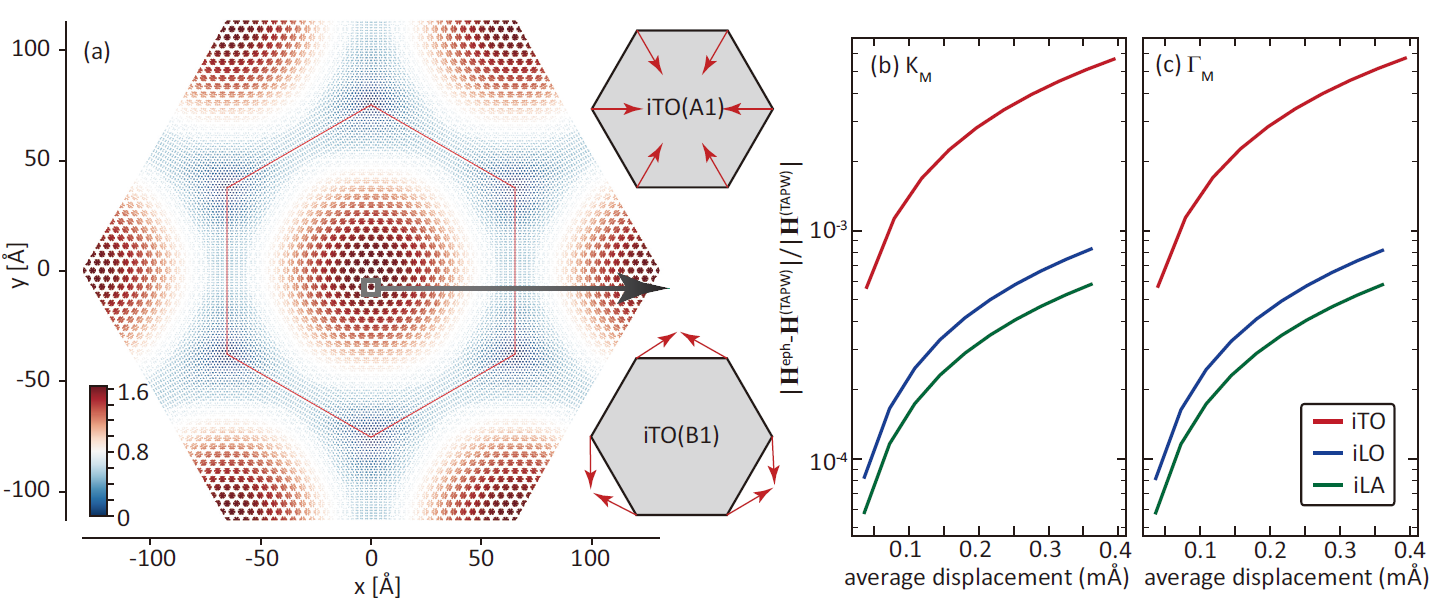


**Fig. S9 | Electron phonon coupling in TBG.** (a) Real space distortion induced by iTO moiré phonons at Γ point. The atomic vibrations are localized at the AA-stacking area and almost zero at the AB-stacking area. The red hexagon marks the Wigner-Seitz cell of MATBG. (b,c) Relative change for the Hamiltonian at K_M_ and Γ_M_ points induced by frozen phonon distortions of iTO (red), iLO (blue), and iLA (green) phonon branches.

Based on the frozen-phonon estimations, we restrict ourselves to the EPC induced by iTO phonons in this paper, although the normal modes of moiré phonons may involve minimal contributions from other ingredients. As shown in panel (a) of Fig. S9, the real space distortion caused by Γ_M_ moiré phonon is localized in the AA-stacking area and obeys all *D_6_* point group symmetry, as the one found in Ref. 23. The localized vibrations behave like atomic iTO-A_1_/iTO-B_1_ mode which are degenerate, as presented in the main text. Moreover, the iTO moiré phonons also display strong inter-valley EPC. Within the frozen-phonon framework, the four flat bands are separated in energy for $\left| \eta\left( r \right) \right|=50mÅ$, as shown in Fig. S10. This energy separation leads to insulating states for all integer electron fillings, which cannot be reproduced by other types of phonons.

The extended BM model considering EPC^24^ written in the continuous limit is

$H\left( \boldsymbol{r} \right)=\left( \begin{matrix} H^{+}(\boldsymbol{r}) & V(\boldsymbol{r}) \\ V^{\dagger}(\boldsymbol{r}) & H^{-}(\boldsymbol{r}) \end{matrix} \right)$ *(7)*

where $H^{\pm}(\boldsymbol{r})$ describes the original BM model for each valley and $V(\boldsymbol{r})$ describes inter valley scattering. In the basis of [A_1_, B_1_, A_2_, B_2_] (A/B is sublattice index and 1/2 is layer index, $V(\boldsymbol{r})$ can be written as

$V\left( \boldsymbol{r} \right)=\left( \begin{matrix} v_{11}(\boldsymbol{r}) & v_{12}(\boldsymbol{r}) \\ v_{21}(\boldsymbol{r}) & v_{22}(\boldsymbol{r}) \end{matrix} \right)$ *(8)*

and

$v_{12}(\boldsymbol{r})=e^{i\boldsymbol{Q\cdot r}}\left( \begin{matrix} 0 & \gamma\\ \gamma& 0 \end{matrix} \right)$, $v_{21}\left( \boldsymbol{r} \right)=e^{i\boldsymbol{Q\cdot r}}\left( \begin{matrix} 0 & \gamma\\ \gamma& 0 \end{matrix} \right)$ *(9)*

$v_{11}(\boldsymbol{r})=e^{i\boldsymbol{Q\cdot r}}\left[ \left( \begin{matrix} g' & g \\ g & g' \end{matrix} \right)+e^{i\boldsymbol{G}_{\boldsymbol{1}}\boldsymbol{\cdot r}}\left( \begin{matrix} \omega g' & g \\ g & \omega^{-1}g' \end{matrix} \right)+e^{-i\boldsymbol{G}_{\boldsymbol{2}}\boldsymbol{\cdot r}}\left( \begin{matrix} \omega^{-1}g' & g \\ g & \omega g' \end{matrix} \right) \right]$ *(10)*

$v_{22}(\boldsymbol{r})=e^{i\boldsymbol{Q\cdot r}}\left[ \left( \begin{matrix} g' & g \\ g & g' \end{matrix} \right)+e^{-i\boldsymbol{G}_{\boldsymbol{1}}\boldsymbol{\cdot r}}\left( \begin{matrix} \omega g' & g \\ g & \omega^{-1}g' \end{matrix} \right)+e^{i\boldsymbol{G}_{\boldsymbol{2}}\boldsymbol{\cdot r}}\left( \begin{matrix} \omega^{-1}g' & g \\ g & \omega g' \end{matrix} \right) \right]$ *(11)*

In the above equations, $\boldsymbol{Q=}\boldsymbol{K}_{\boldsymbol{1}}^{\boldsymbol{+}}\boldsymbol{-}\boldsymbol{K}_{\boldsymbol{2}}^{\boldsymbol{-}}$， $\omega=e^{i2\pi/3}$. The matrix form of EPC matrix *M_0_* in the atomic momentum space is

$M_{0}=\left[ \begin{matrix} 0 & \boldsymbol{V} \\ \boldsymbol{V}^{\boldsymbol{\dagger}} & 0 \end{matrix} \right]$ *(12)*

$\boldsymbol{V=}\left[ \begin{matrix} \boldsymbol{*} & \boldsymbol{*} & \cdots& \boldsymbol{*} & V' & 0 & \cdots& 0 \\ \boldsymbol{*} & \boldsymbol{*} & \cdots& \boldsymbol{*} & 0 & V' & \cdots& 0 \\ \vdots& \vdots& \ddots& \cdots& \vdots& \vdots& \ddots& \cdots\\ \boldsymbol{*} & \boldsymbol{*} & \vdots& \boldsymbol{*} & 0 & 0 & \vdots& V' \\ V' & 0 & \cdots& 0 & ⋄ & ⋄ & \cdots& ⋄ \\ 0 & V' & \cdots& 0 & ⋄ & ⋄ & \cdots& ⋄ \\ \vdots& \vdots& \ddots& \cdots& \vdots& \vdots& \ddots& \cdots\\ 0 & 0 & \vdots& V' & ⋄ & ⋄ & \vdots& ⋄ \end{matrix} \right]$ *(13)*

where

$\boldsymbol{*=}\left\langle\boldsymbol{G}_{\boldsymbol{n}} | V | \boldsymbol{G}_{\boldsymbol{m}} \right\rangle\boldsymbol{=}\left\{ \begin{matrix} V_{0}, if \boldsymbol{G}_{\boldsymbol{m}}\boldsymbol{-}\boldsymbol{G}_{\boldsymbol{n}}\boldsymbol{=0} \\ V_{1}, if \boldsymbol{G}_{\boldsymbol{m}}\boldsymbol{-}\boldsymbol{G}_{\boldsymbol{n}}\boldsymbol{=}\boldsymbol{G}_{\boldsymbol{1}} \\ V_{2}, if \boldsymbol{G}_{\boldsymbol{m}}\boldsymbol{-}\boldsymbol{G}_{\boldsymbol{n}}\boldsymbol{=}\boldsymbol{-G}_{\boldsymbol{2}} \\ \boldsymbol{0}, else \end{matrix} \right.$ *(14)*

$⋄\boldsymbol{=}\left\langle\boldsymbol{G}_{\boldsymbol{n}} | V | \boldsymbol{G}_{\boldsymbol{m}} \right\rangle\boldsymbol{=}\left\{ \begin{matrix} V_{0}, if \boldsymbol{G}_{\boldsymbol{m}}\boldsymbol{-}\boldsymbol{G}_{\boldsymbol{n}}\boldsymbol{=0} \\ V_{1}, if \boldsymbol{G}_{\boldsymbol{m}}\boldsymbol{-}\boldsymbol{G}_{\boldsymbol{n}}\boldsymbol{=}\boldsymbol{-G}_{\boldsymbol{1}} \\ V_{2}, if \boldsymbol{G}_{\boldsymbol{m}}\boldsymbol{-}\boldsymbol{G}_{\boldsymbol{n}}\boldsymbol{=}\boldsymbol{G}_{\boldsymbol{2}} \\ \boldsymbol{0}, else \end{matrix} \right.$ *(15)*

$V^{'}=\left[ \begin{matrix} 0 & \gamma\\ \gamma& 0 \end{matrix} \right], V_{0}=\left[ \begin{matrix} g' & g \\ g & g' \end{matrix} \right], V_{1}=\left[ \begin{matrix} \omega g' & g \\ g & \omega^{-1}g' \end{matrix} \right],V_{2}=\left[ \begin{matrix} g\omega^{-1} & g \\ g & \omega g' \end{matrix} \right]$ *(16)*

and $\omega=e^{i2\pi/3}$.


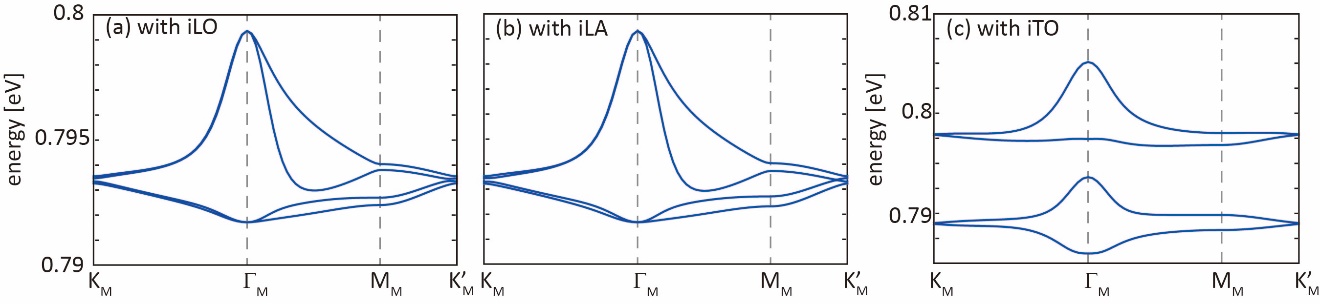


**Fig. S10 | Frozen phonon calculation.** Band structure affected by frozen phonons for (a) iLO type, (b) iLA type, and (c) iTO type. The distortion field $\eta\left( r \right)$ is constrained by |$\eta\left( r \right)|=50mÅ$. The flat bands from two atomic valleys are totally gapped out when iTO moiré phonon is frozen, which indicates strong cross valley EPC.

Our TAPW method can help determine the effective parameters of the extended BM model when performing plane-wave projection, as illustrated in Fig. S11. Related technique details are discussed in Ref. 19, 24&25. These parameters are summarized in Table 1: $u,u'$ denote the intra-valley, inter-layer coupling strengths (parameters in original BM model); $g,g'$ are inter-valley, intra-layer coupling strengths for different sublattices, respectively; $\gamma$ is inter-valley, inter-layer coupling strength. In our frozen-phonon calculation, $g,g^{'},\gamma$ depend linearly with $|\eta\left( r \right)|$ while $u,u'$ are independent. It suggests the iTO moiré phonon only contribute to inter-valley couplings, absent in the original BM model. Using these linearly dependent parameters, we can evaluate the effective EPC matrix $M_{0}$, which is the inter-valley part of the Hamiltonian (can be built using the method described in Ref. 19 or directly read from TAPW method Ref. 24 & 25) when $|\eta\left( r \right)|$ is confined at characteristic phonon length, with second quantized phonons. We set the moiré phonon energy $\omega_{0}=150\mathrm{meV}$ and the mass of carbon atoms $m_{c}=2.0\times{10}^{-26}\mathrm{kg}$, and then the characteristic phonon length should be $l_{p}= \sqrt{\hbar/(2m_{c}\omega_{0})}=34.0 mÅ$. After fixing $|\eta\left( r \right)|$ to $l_{p}$, we can retrieve the normalized EPC parameters in $M_{0}$: $\bar{g}=3.1882 \mathrm{meV},\bar{g'}=0.2569 \mathrm{meV}$, and $\bar{\gamma}=0.07611 \mathrm{meV}$.

With the parameters extracted from frozen-phonon simulations, we write both the electrons and phonons in the moiré-BZ for folded TBG systems. We denote the size of moiré mini-BZ as $N_{m}$ for an *N*-site system. The general Hamiltonian of TBG bands and the linear EPC reads

$$H= \sum_{\bar{\boldsymbol{k}}\sigma} \boldsymbol{c}_{\bar{\boldsymbol{k}}\sigma}^{\left( \mathrm{TAPW} \right)\dagger}h_{\bar{\boldsymbol{k}}}\boldsymbol{c}_{\bar{\boldsymbol{k}}\sigma}^{\left( \mathrm{TAPW} \right)}-\frac{1}{\sqrt{N_{m}}}\sum_{\bar{\boldsymbol{k}}\sigma\bar{\boldsymbol{q}}\nu} \boldsymbol{c}_{\bar{\boldsymbol{k}}+\bar{\boldsymbol{q}}\sigma}^{\left( \mathrm{TAPW} \right)\dagger}M_{\bar{\boldsymbol{q}}\nu}\boldsymbol{c}_{\bar{\boldsymbol{k}}\sigma}^{\left( \mathrm{TAPW} \right)}\left( a_{\bar{\boldsymbol{q}}\nu}+a_{-\bar{\boldsymbol{q}}\nu}^{\dagger} \right)+\sum_{\bar{\boldsymbol{q}}\nu} \omega_{0}a_{\bar{\boldsymbol{q}}\nu}^{\dagger}a_{\bar{\boldsymbol{q}}\nu}$$

*(17)*

where $\boldsymbol{c}_{\bar{\boldsymbol{k}}\sigma}^{\left( \mathrm{TAPW} \right)}$ is the column vector of electron annihilation operators with moiré momentum $\bar{\boldsymbol{k}}$ and spin $\sigma$ in the TAPW basis (representing the hyper-index ($\boldsymbol{G}\alpha$). Here, $\boldsymbol{G}$ is moiré reciprocal lattice vector and $\alpha\in\{A_{1},B_{1},A_{2},B_{2}\}$ with $A, B$ for atoms in the primitive cell of graphene and the subscripts for two layers) and $h_{\bar{\boldsymbol{k}}}$ is the electronic hopping matrix for a specific moiré momentum $\bar{\boldsymbol{k}}$. Together with electrons, the phonon modes are also folded into the mini-BZ, accompanied by the increase of branches. Here, the $a_{\bar{\boldsymbol{q}}\nu}$ is the phonon annihilation operator with moiré momentum $\bar{\boldsymbol{q}}$ and $\nu$ labels the folded branches and $\omega_{0}$ is the phonon frequency, assumed to be Einstein phonon within the moiré-BZ. The coupling matrix between electrons and phonons is denoted as $M_{\bar{\boldsymbol{q}}\nu}$, whose long-wavelength limit is the $M_{0}$ mentioned above. Note that the TBG Hamiltonian in Eq. (17) does not take hBN substrates into account, reflecting the unaligned situations where the hBN potential is incommensurate with moiré supercells and smears out in the spatial average. The impact of commensurate (aligned) hBN potential will be discussed in Sec. X.


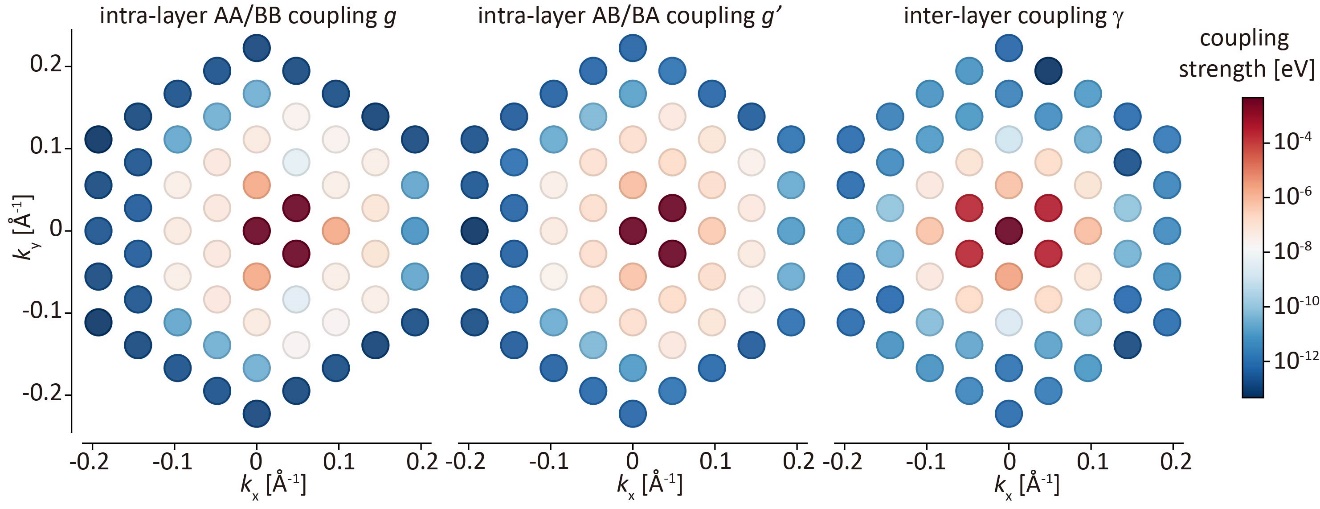


**Fig. S11 |** **Determining effective parameters in** **extended BM model using TAPW method.** The plane wave components analysis of the inter-valley coupling Hamiltonian gives us three effective parameters to extend the original BM model. Each small circle represents an atomic plane wave, and it presents good convergence of our numerical results. $g,g'$ are parameters for inter-valley, intra-layer coupling for two sets of sublattices. $\gamma$ is the parameter for inter-valley, inter-layer coupling.

**Table 1 | Effective parameters extracted from the TAPW method**

| $\boldsymbol{\vert}\boldsymbol{\eta}\left( \boldsymbol{r} \right)\boldsymbol{\vert} \mathbf{(}\mathbf{m}\mathbf{Å}\mathbf{)}$ | $\boldsymbol{u} \mathbf{(meV)}$ | $\boldsymbol{u}^{\boldsymbol{'}}\mathbf{(meV)}$ | $\boldsymbol{g}\mathbf{(meV)}$ | $\boldsymbol{g'}\mathbf{(meV)}$ | $\boldsymbol{\gamma}\mathbf{(meV)}$ |
| --- | --- | --- | --- | --- | --- |
| 5.00 | 85.81 | 103.2 | 0.4685 | 0.03758 | 0.01114 |
| 10.0 | 85.81 | 103.2 | 0.9379 | 0.07517 | 0.02228 |
| 20.0 | 85.81 | 103.2 | 1.873 | 0.1503 | 0.04455 |
| 30.0 | 85.81 | 103.2 | 2.810 | 0.2255 | 0.06681 |
| 40.0 | 85.81 | 103.2 | 3.746 | 0.3007 | 0.08906 |
| 50.0 | 85.81 | 103.2 | 4.682 | 0.3759 | 0.1111 |

We gauge the strength of coupling using the dimensionless EPC parameter $\lambda_{\mathrm{Eliashberg}}=2\int_{0}^{\infty} \frac{\alpha^{2}F\left( \omega\right)}{\omega}d\omega$, where $\alpha^{2}F\left( \omega\right)$ is the Eliashberg phonon spectral function. When neglecting phonon dispersions and the momentum dispersion of the coupling matrix elements – which is a good approximation for the optical phonons in the current case – this parameter can be reduced to

$$\lambda_{\mathrm{Eliashberg}}=2\rho_{F}\sum_{\nu} \frac{g_{v}^{2}}{\omega_{v}}$$

where $g_{\nu}$，$\omega_{\nu}$ represent the coupling strength and the mode energy for the $v^{th}$-branch phonon in the folded Brilliouin zone (BZ), respectively. $\rho_{F}$denotes the density of states at the Fermi-energy, which is inversely proportional to the bandwidth. Given the small bandwidth (~10 meV) of the flat band electrons, we obtain $\lambda_{\mathrm{Eliashberg}}=13\gg1$, indicating a strong-coupling scenario. This convention is commonly used, as the strength of $\lambda_{\mathrm{Eliashberg}}$ determines the validity of perturbation theory (see e.g. Ref. 26). The large $\lambda_{\mathrm{Eliashberg}}$ in MATBG arises from the presence of the flat-band, similar to the strong correlation effects for electrons. Due to the strong coupling nature, the Migdal-Eliashberg theory is no longer reliable, and we have to employ the variational method discussed in this work.

**IX. Quantum phonon dressing and ARPES spectra in TBG**

We will simulate the single-particle spectrum for bare TBG without hBN alignment in this section. To simplify the system, we project the full Hamiltonian from the TAPW orbitals onto the flat-band orbitals, defining a column of annihilation operators with four elements (two valleys and two bands) $\boldsymbol{c}_{\bar{\boldsymbol{k}}\sigma}^{\left( \mathrm{flat} \right)}=P_{\bar{\boldsymbol{k}}}^{\dagger}\boldsymbol{c}_{\bar{\boldsymbol{k}}\sigma}^{\left( \mathrm{TAPW} \right)}$. These bands are chosen as eigenstates of the electronic kinetic Hamiltonian, i.e. $P_{\bar{\boldsymbol{k}}}^{\dagger}h_{\bar{\boldsymbol{k}}}P_{\bar{\boldsymbol{k}}}$ is diagonal. Note that the combination of the two bands on each valley forms the chiral basis, where the EPC matrix is strictly intervalley^27^. Therefore, the projected EPC matrix $\tilde{M}_{\bar{\boldsymbol{k}}\bar{\boldsymbol{q}}\nu}={P_{\bar{\boldsymbol{k}}\boldsymbol{+}\bar{\boldsymbol{q}}}^{\dagger}M}_{\bar{\boldsymbol{q}}\nu}P_{\bar{\boldsymbol{k}}}$ is also restricted to cross-valley couplings.

The coupling matrix with both $\bar{\boldsymbol{k}}$ and $\bar{\boldsymbol{q}}$dependence is difficult to solve analytically. Since the $\bar{\boldsymbol{q}}$dependence of $\tilde{M}_{\bar{\boldsymbol{k}}\bar{\boldsymbol{q}}\nu}$ is weak, we focus on the $\bar{\boldsymbol{q}}\boldsymbol{=0}$ situation first. With this assumption, we can find an SU(4) transformation that diagonalizes the inter-valley coupling, i.e. $U_{\bar{\boldsymbol{k}}}^{\dagger}\tilde{M}_{\bar{\boldsymbol{k}}\bar{\boldsymbol{q}}\nu}U_{\bar{\boldsymbol{k}}}=\mathrm{diag}(g_{\bar{\boldsymbol{k}}}^{\left( 1 \right)},g_{\bar{\boldsymbol{k}}}^{\left( 2 \right)},g_{\bar{\boldsymbol{k}}}^{\left( 3 \right)},g_{\bar{\boldsymbol{k}}}^{(4)})$. Based on our numerical simulations, we observe two consequences. First, due to the identical chemical environment of these four flat bands, the band-specified coupling strength $g_{\bar{\boldsymbol{k}}}^{\left( \alpha\right)}$ are identical to each other, with only a difference of parity (two positive ones and two negative ones). That being said, the coupling strength can be written as $g_{\bar{\boldsymbol{k}}}\eta$, $\eta=\mathrm{diag}(1,1,-1,-1)$. Second, we notice that the spatial distribution of $g\left( \bar{\boldsymbol{R}} \right)=\frac{1}{N_{m}}\sum_{\bar{\boldsymbol{k}}} e^{-i\bar{\boldsymbol{R}}\cdot\bar{\boldsymbol{k}}}g_{\bar{\boldsymbol{k}}}$ is highly localized at $\bar{\boldsymbol{R}}\boldsymbol{=}\bar{\boldsymbol{0}}$, indicating that the variation of EPC is almost canceled out within the sizeable moiré electronic wavefunctions. Thus, one can further drop the momentum dependence of the coupling matrix $g_{\bar{\boldsymbol{k}}}\approx g$. After these simplifications in the EPC-diagonal basis, we can recapture the $\bar{\boldsymbol{q}}$-dependence of the coupling matrix, we further approximate $g_{\bar{\boldsymbol{q}}}\approx\frac{1}{N_{m}}\sum_{\bar{\boldsymbol{k}}} |\det\left( P_{\bar{\boldsymbol{k}}\boldsymbol{+}\bar{\boldsymbol{q}}}^{\dagger}M_{\bar{\boldsymbol{q}}}P_{\bar{\boldsymbol{k}}} \right)\left. \right|^{1/4}$. The calculated $g_{\bar{\boldsymbol{q}}}$ via frozen-phonon method varies from 4.1 meV to 5.2 meV. The small variation of $g_{\bar{\boldsymbol{q}}}$ is consistent with our assumption that the coupling matrix is nearly independent with $\bar{\boldsymbol{q}}$. Therefore, using the above observations and approximations, we simplify the Hamiltonian by rotating the flat-band eigen basis to the EPC-diagonal basis $\boldsymbol{d}_{\bar{\boldsymbol{k}}\sigma}=Q_{\bar{\boldsymbol{k}}}^{\dagger}\boldsymbol{c}_{\bar{\boldsymbol{k}}\sigma}^{\left( \mathrm{flat} \right)}$, that is

$H= \sum_{\bar{\boldsymbol{k}}\sigma} \boldsymbol{d}_{\bar{\boldsymbol{k}}\sigma}^{\dagger}\tilde{h}(\bar{\boldsymbol{k}})\boldsymbol{d}_{\bar{\boldsymbol{k}}\sigma}-\frac{1}{\sqrt{N_{m}}}\sum_{\bar{\boldsymbol{k}}\sigma\bar{\boldsymbol{q}}\nu} {g_{\bar{\boldsymbol{q}}}\boldsymbol{d}}_{\bar{\boldsymbol{k}}+\bar{\boldsymbol{q}}\sigma}^{\dagger}\eta\boldsymbol{d}_{\bar{\boldsymbol{k}}\sigma}\left( a_{\bar{\boldsymbol{q}}\nu}+a_{-\bar{\boldsymbol{q}}\nu}^{\dagger} \right)+\sum_{\bar{\boldsymbol{q}}\nu} \omega_{0}a_{\bar{\boldsymbol{q}}\nu}^{\dagger}a_{\bar{\boldsymbol{q}}\nu}$ *(18)*

where $\tilde{h}(\bar{\boldsymbol{k}})=Q_{\bar{\boldsymbol{k}}}^{\dagger}\varepsilon_{\bar{\boldsymbol{k}}}Q_{\bar{\boldsymbol{k}}}$ contains off-diagonal hopping terms between different bands since the transformed electronic basis are no longer the eigen-basis for the kinetic Hamiltonian. The characteristic energy of $\tilde{h}(\bar{\boldsymbol{k}})$ is within 5 meV and the phonon energy is set to $\omega_{0}=150 \mathrm{meV}$.

Due to the strong coupling and high phonon frequency, compared to the electronic bandwidth, the polaronic dressing effect cannot be treated as a perturbation. Instead, we consider the Lang-Firsov transformation for the coupled Hamiltonian^28^

$U_{\mathrm{LF}}=\exp\left[ \frac{1}{\sqrt{N_{m}}\omega_{0}}\sum_{\bar{\boldsymbol{R}}\sigma\bar{\boldsymbol{q}}\nu} e^{-i\bar{\boldsymbol{R}}\cdot\bar{\boldsymbol{q}}}{g_{\bar{\boldsymbol{q}}}\boldsymbol{d}}_{\bar{\boldsymbol{R}}\sigma}^{\dagger}\eta\boldsymbol{d}_{\bar{\boldsymbol{R}}\sigma}\left( a_{\bar{\boldsymbol{q}}\nu}-a_{-\bar{\boldsymbol{q}}\nu}^{\dagger} \right) \right]$ *(19)*

and the transformed annihilation operators are

$U_{\mathrm{LF}}c_{\bar{\boldsymbol{R}}\sigma}U_{\mathrm{LF}}^{\dagger}=\exp\left[ -\frac{1}{\sqrt{N_{m}}\omega_{0}}\sum_{\bar{\boldsymbol{q}}\nu} e^{-i\bar{\boldsymbol{R}}\cdot\bar{\boldsymbol{q}}}g_{\bar{\boldsymbol{q}}}\left( a_{\bar{\boldsymbol{q}}\nu}-a_{-\bar{\boldsymbol{q}}\nu}^{\dagger} \right)\eta\right]c_{\bar{\boldsymbol{R}}\sigma}$ *(20)*

$U_{\mathrm{LF}}a_{\bar{\boldsymbol{q}}\nu}U_{\mathrm{LF}}^{\dagger}=a_{\bar{\boldsymbol{q}}\nu}+\frac{1}{\sqrt{N_{m}}\omega_{0}}\sum_{\bar{\boldsymbol{R}}\sigma} e^{i\bar{\boldsymbol{R}}\cdot\bar{\boldsymbol{q}}}g_{-\bar{\boldsymbol{q}}}\boldsymbol{d}_{\bar{\boldsymbol{R}}\sigma}^{\dagger}\eta\boldsymbol{d}_{\bar{\boldsymbol{R}}\sigma}$ *(21)*

where $\boldsymbol{c}_{\bar{\boldsymbol{R}}\sigma}$ and $a_{\bar{\boldsymbol{q}}\nu}$ are annihilation operators of polaron (for sublattices and bands as the column indices) and distorted phonon, respectively. The transformed Hamiltonian $H$ can be rewritten as

$$U_{\mathrm{LF}}HU_{\mathrm{LF}}^{\dagger}=\sum_{\bar{\boldsymbol{R}}\bar{\boldsymbol{R}^{\boldsymbol{'}}}\sigma} \boldsymbol{d}_{\bar{\boldsymbol{R}}\sigma}^{\dagger}h_{\bar{\boldsymbol{R}}\bar{\boldsymbol{R}^{\boldsymbol{'}}}}^{*}\boldsymbol{d}_{\bar{\boldsymbol{R}^{\boldsymbol{'}}}\sigma}-\sum_{\bar{\boldsymbol{R}}\bar{\boldsymbol{R}^{\boldsymbol{'}}}\sigma\sigma^{'}\bar{\boldsymbol{q}}} \frac{N_{v}\left| g_{\bar{\boldsymbol{q}}} \right|^{2}}{N_{m}\omega_{0}}e^{i\left( \bar{\boldsymbol{R}^{\boldsymbol{'}}}\boldsymbol{-}\bar{\boldsymbol{R}} \right)\cdot\bar{\boldsymbol{q}}}\left( \boldsymbol{d}_{\bar{\boldsymbol{R}}\sigma}^{\dagger}\eta\boldsymbol{d}_{\bar{\boldsymbol{R}}\sigma} \right)\left( \boldsymbol{d}_{\bar{\boldsymbol{R}^{\boldsymbol{'}}}\sigma^{'}}^{\dagger}\eta\boldsymbol{d}_{\bar{\boldsymbol{R}^{\boldsymbol{'}}}\sigma^{'}} \right)+\sum_{\bar{\boldsymbol{q}}v} \omega_{0}a_{\bar{\boldsymbol{q}}v}^{\dagger}a_{\bar{\boldsymbol{q}}v}$$

*(22)*

where the EPC is transformed into the kinetic energy terms with

$h_{\bar{\boldsymbol{R}}\bar{\boldsymbol{R}^{\boldsymbol{'}}}}^{*}=\exp\left[ \frac{g}{\sqrt{N_{m}}\omega_{0}}\sum_{\bar{\boldsymbol{q}}\nu} e^{-i\bar{\boldsymbol{R}}\cdot\bar{\boldsymbol{q}}}\left( a_{\bar{\boldsymbol{q}}\nu}-a_{-\bar{\boldsymbol{q}}\nu}^{\dagger} \right)\eta\right]\tilde{h}_{\bar{\boldsymbol{R}}\bar{\boldsymbol{R}^{\boldsymbol{'}}}}\exp\left[ -\frac{g}{\sqrt{N_{m}}\omega_{0}}\sum_{\bar{\boldsymbol{q}}\nu} e^{-i\bar{\boldsymbol{R}}\cdot\bar{\boldsymbol{q}}}\left( a_{\bar{\boldsymbol{q}}\nu}-a_{-\bar{\boldsymbol{q}}\nu}^{\dagger} \right)\eta\right]$ *(23)*

in which $\tilde{h}_{\bar{\boldsymbol{R}}\bar{\boldsymbol{R}^{\boldsymbol{'}}}}=\frac{1}{N_{m}}\sum_{\bar{\boldsymbol{k}}} e^{-i(\bar{\boldsymbol{R}}-\bar{\boldsymbol{R}^{\boldsymbol{'}}})\cdot\bar{\boldsymbol{k}}}\tilde{h}(\bar{\boldsymbol{k}})$. Since this term is bounded by the electronic bandwidth, which is smaller than the EPC induced interaction and phonon energy $\omega_{0}$, we assume that its ground state (and low-energy excited states) can be regarded as an electron-phonon separable state $|\left. \tilde{\Psi} \right\rangle=|\left. \psi_{e} \right\rangle\bigotimes|\left. \psi_{\mathrm{ph}} \right\rangle$. That being said, the ground-state wavefunction for the original Hamiltonian Eq. (18) is $|\left. \Psi_{G} \right\rangle=U_{\mathrm{LF}}^{\dagger}|\left. \psi_{e} \right\rangle\bigotimes|\left. \psi_{\mathrm{ph}} \right\rangle$.

Moreover, since both the transformed coupling strength and temperature are much less than the phonon energy $\omega\sim150 \mathrm{meV}$, we further assume that the $|\left. \psi_{\mathrm{ph}} \right\rangle$ can be approximated by a vacuum state $|\left. 0_{\mathrm{ph}} \right\rangle$. Thus, we can project the transformed Hamiltonian Eq. (22) onto the phonon vacuum state and obtain the effective electronic Hamiltonian

$H_{e}=\left\langle0_{\mathrm{ph}} \right.\left| U_{\mathrm{LF}}HU_{\mathrm{LF}}^{\dagger} \right|\left. 0_{\mathrm{ph}} \right\rangle=\sum_{\bar{\boldsymbol{R}}\bar{\boldsymbol{R}^{\boldsymbol{'}}}\sigma} \boldsymbol{d}_{\bar{\boldsymbol{R}}\sigma}^{\dagger}\left\langle0_{\mathrm{ph}} \right.\left| h_{\bar{\boldsymbol{R}}\bar{\boldsymbol{R}^{\boldsymbol{'}}}}^{*} \right|\left. 0_{\mathrm{ph}} \right\rangle\boldsymbol{d}_{\bar{\boldsymbol{R}^{\boldsymbol{'}}}\sigma}-\sum_{\bar{\boldsymbol{R}}\bar{\boldsymbol{R}^{\boldsymbol{'}}}\sigma\sigma^{'}\bar{\boldsymbol{q}}} \frac{N_{v}\left| g_{\bar{\boldsymbol{q}}} \right|^{2}}{N_{m}\omega_{0}}e^{i\left( \bar{\boldsymbol{R}^{\boldsymbol{'}}}\boldsymbol{-}\bar{\boldsymbol{R}} \right)\cdot\bar{\boldsymbol{q}}}\left( \boldsymbol{d}_{\bar{\boldsymbol{R}}\sigma}^{\dagger}\eta\boldsymbol{d}_{\bar{\boldsymbol{R}}\sigma} \right)\left( \boldsymbol{d}_{\bar{\boldsymbol{R}^{\boldsymbol{'}}}\sigma^{'}}^{\dagger}\eta\boldsymbol{d}_{\bar{\boldsymbol{R}^{\boldsymbol{'}}}\sigma^{'}} \right)$ *(24)*

whose ground state determines the $|\left. \psi_{e} \right\rangle$ in our wavefunction ansatz. The on-site $(\bar{\boldsymbol{R}}=\bar{\boldsymbol{R}^{\boldsymbol{'}}})$ term with coupling strength $U=\sum_{\bar{\boldsymbol{q}}} \frac{N_{v}\left| g_{\bar{\boldsymbol{q}}} \right|^{2}}{N_{m}\omega_{0}}=16.5 \mathrm{meV}$ dominates the electron-electron interaction induced by the $\left| g_{\bar{\boldsymbol{q}}} \right|^{2}$ term of Eq. (24).

Spectral properties of the system are not only encoded by the ground-state wavefunction, but also lie in the excited states. Specifically, the photoemission spectrum can be written as

$A\left( \bar{\boldsymbol{k}},\omega\right)=\mathrm{Im}\left\{ \frac{1}{N_{m}}\sum_{\bar{\boldsymbol{R}}\bar{\boldsymbol{R}^{\boldsymbol{'}}}} e^{-i\left( \bar{\boldsymbol{R}}\boldsymbol{-}\bar{\boldsymbol{R}^{\boldsymbol{'}}} \right)\cdot\bar{\boldsymbol{k}}}G_{\bar{\boldsymbol{R}}\bar{\boldsymbol{R}^{\boldsymbol{'}}}}(\omega-i\Gamma) \right\}$ *(25)*

where $\Gamma$ is the Lorentzian broadening and the electron Green’s function is

$G_{\bar{\boldsymbol{R}}{\bar{\boldsymbol{R}}}^{'}}\left( z \right)=\sum_{\sigma\alpha, excited states \Phi} \left\langle\Psi_{G} \right.\left| U_{\bar{\boldsymbol{k}}}^{\dagger}d_{\bar{\boldsymbol{R}}\sigma\alpha}^{\dagger} \right|\left. \Phi\right\rangle\left\langle\Phi\right.\left| U_{\bar{\boldsymbol{k}}}d_{\bar{\boldsymbol{R}^{\boldsymbol{'}}}\sigma\alpha} \right|\left. \Psi_{G} \right\rangle\frac{1}{z+E_{\Phi}-E_{G}}$ . *(26)*

Considering final state $|\left. \Phi\right\rangle$ belongs to a many-body state with $N_{e}-1$ electrons and the Lang-Firsov transformation $U_{LF}$ conserves particle number, the amplitude can be expressed in the polaronic basis using the wavefunction ansatz. Then we obtain

$$\left\langle\Phi\right.\left| c_{\bar{\boldsymbol{R}}\sigma\alpha} \right|\left. \Psi_{G} \right\rangle=\left\langle\psi_{\mathrm{ph}}\left\{ m_{\bar{\boldsymbol{q}}\nu} \right\} \right.\left| \bigotimes\left\langle\psi_{e}^{N_{e}-1} \right.\left| U_{\mathrm{LF}}c_{\bar{\boldsymbol{R}}\sigma\alpha}U_{\mathrm{LF}}^{\dagger} \right|\left. \psi_{e}^{N_{e}} \right\rangle\right|\left. 0_{ph} \right\rangle$$

$= \left\langle\psi_{e}^{N_{e}-1} \right.\left| c_{\bar{\boldsymbol{R}}\sigma\alpha} \right|\left. \psi_{e}^{N_{e}} \right\rangle\left\langle\psi_{\mathrm{ph}}\left\{ m_{\bar{\boldsymbol{q}}\nu} \right\} \right.\left| \exp\left[ -\frac{1}{\sqrt{N_{m}}\omega_{0}}\sum_{\bar{\boldsymbol{q}}\nu} e^{-i\bar{\boldsymbol{R}}\cdot\bar{\boldsymbol{q}}}g_{\bar{\boldsymbol{q}}}\left( a_{\bar{\boldsymbol{q}}\nu}-a_{-\bar{\boldsymbol{q}}\nu}^{\dagger} \right)\eta\right] \right|\left. 0_{ph} \right\rangle$.

*(27)*

Since the phonon final states with the same total phonon numbers $M$ share the same energy $M\omega_{0}$ the phonon part of the cross-section can be merged into

$A_{ph}^{\left( M \right)}\left( \bar{\boldsymbol{R}}-\bar{\boldsymbol{R}^{\boldsymbol{'}}} \right)=\sum_{\sum m_{\bar{\boldsymbol{q}}\nu}=M} \left\langle0_{\mathrm{ph}} \right.\left| \exp\left[ \frac{1}{\sqrt{N_{m}}\omega_{0}}\sum_{\bar{\boldsymbol{q}}\nu} e^{-i\bar{\boldsymbol{R}}\cdot\bar{\boldsymbol{q}}}g_{\bar{\boldsymbol{q}}}\left( a_{\bar{\boldsymbol{q}}\nu}-a_{-\bar{\boldsymbol{q}}\nu}^{\dagger} \right)\eta\right] \right|\left. \psi_{\mathrm{ph}}\left\{ m_{\bar{\boldsymbol{q}}\nu} \right\} \right\rangle\times\left\langle\psi_{\mathrm{ph}}\left\{ m_{\bar{\boldsymbol{q}}\nu} \right\} \right.\left| \exp\left[ -\frac{1}{\sqrt{N_{m}}\omega_{0}}\sum_{\bar{\boldsymbol{q}}\nu} e^{-i\bar{\boldsymbol{R}}\cdot\bar{\boldsymbol{q}}}g_{\bar{\boldsymbol{q}}}\left( a_{\bar{\boldsymbol{q}}\nu}-a_{-\bar{\boldsymbol{q}}\nu}^{\dagger} \right)\eta\right] \right|\left. 0_{\mathrm{ph}} \right\rangle=\sum_{\sum m_{\bar{\boldsymbol{q}}\nu}=M} \prod_{\bar{\boldsymbol{q}}\nu} \exp\left( -\frac{\left| g_{\bar{\boldsymbol{q}}} \right|^{2}}{N_{m}\omega_{0}^{2}} \right)\frac{1}{\left( m_{\bar{\boldsymbol{q}}\nu} \right)!}\left( \frac{\left| g_{\bar{\boldsymbol{q}}} \right|^{2}}{N_{m}\omega_{0}^{2}}e^{i(\bar{\boldsymbol{R}^{\boldsymbol{'}}}-\bar{\boldsymbol{R}})\cdot\bar{\boldsymbol{q}}} \right)^{m_{\bar{\boldsymbol{q}}\nu}}=\frac{1}{M!}\exp\left( -\sum_{\bar{\boldsymbol{q}}} \frac{N_{v}\left| g_{\bar{\boldsymbol{q}}} \right|^{2}}{N_{m}\omega_{0}^{2}} \right)\left( \sum_{\bar{\boldsymbol{q}}} \frac{N_{v}\left| g_{\bar{\boldsymbol{q}}} \right|^{2}}{N_{m}\omega_{0}^{2}}e^{i(\bar{\boldsymbol{R}}-\bar{\boldsymbol{R}^{\boldsymbol{'}}})\cdot\bar{\boldsymbol{q}}} \right)^{M}$ *(28)*

We approximate $|\left. \psi_{e}^{N_{e}} \right\rangle$ by the ground-state of $H_{e}$ in the non-interacting limit

$H_{e}^{non-int}= \sum_{\bar{\boldsymbol{R}}\bar{\boldsymbol{R}^{\boldsymbol{'}}}\sigma} c_{\bar{\boldsymbol{R}}\sigma}^{\dagger}\left\langle0_{\mathrm{ph}} \right.\left| h_{\bar{\boldsymbol{R}}\bar{\boldsymbol{R}^{\boldsymbol{'}}}}^{*} \right|\left. 0_{\mathrm{ph}} \right\rangle c_{\bar{\boldsymbol{R}^{\boldsymbol{'}}}\sigma}=\sum_{\bar{\boldsymbol{k}}\sigma} \boldsymbol{c}_{\bar{\boldsymbol{k}}\sigma}^{\dagger}h_{\bar{\boldsymbol{k}}}^{*}\boldsymbol{c}_{\bar{\boldsymbol{k}^{\boldsymbol{'}}}\sigma}$ *(29)*

where the hopping matrix $h_{\bar{\boldsymbol{k}}}^{*}$ takes the explicit form

$h_{\bar{\boldsymbol{k}}\boldsymbol{,}\alpha\alpha^{'}}^{*}=\frac{1}{N_{m}}\sum_{\bar{\boldsymbol{R}}\bar{\boldsymbol{R}^{\boldsymbol{'}}}} e^{-i\left( \bar{\boldsymbol{R}}\boldsymbol{-}\bar{\boldsymbol{R}^{\boldsymbol{'}}} \right)}\exp\left\{ -\frac{N_{v}}{\omega_{0}^{2}N_{m}}\sum_{\bar{\boldsymbol{q}}} g_{\bar{\boldsymbol{q}}}^{2}\left[ 1-\eta_{\alpha\alpha}\eta_{\alpha^{'}\alpha^{'}}\cos\bar{\boldsymbol{q}}\cdot\left( \bar{\boldsymbol{R}}\boldsymbol{-}\bar{\boldsymbol{R}^{\boldsymbol{'}}} \right) \right] \right\}\tilde{h}_{\bar{\boldsymbol{R}}\bar{\boldsymbol{R}^{\boldsymbol{'}}},\alpha\alpha^{'}}$ *(30)*

Combining the results above, the photoemission spectrum can be simplified as

$A\left( \bar{\boldsymbol{k}},\omega\right)=\sum_{M=0}^{\infty} \mathrm{Im}\left\{ \frac{1}{N_{m}}\sum_{\bar{\boldsymbol{R}}\bar{\boldsymbol{k'}}\alpha} A_{\mathrm{ph}}^{\left( M \right)}(\bar{\boldsymbol{R}})e^{-i\bar{\boldsymbol{R}\cdot}\left( \bar{\boldsymbol{k}}\boldsymbol{-}\bar{\boldsymbol{k}^{\boldsymbol{'}}} \right)}\frac{\theta\left[ \varepsilon_{F}-\varepsilon_{\alpha}^{*}(\bar{\boldsymbol{k}^{\boldsymbol{'}}}) \right]}{\omega-\varepsilon_{\alpha}^{*}\left( \bar{\boldsymbol{k}^{\boldsymbol{'}}} \right)+\varepsilon_{F}+M\omega_{0}-i\Gamma} \right\}$ *(31)*

where $\varepsilon_{\alpha}^{*}\left( \bar{\boldsymbol{k}^{\boldsymbol{'}}} \right)$are eigenvalues of $h_{\bar{\boldsymbol{k}^{\boldsymbol{'}}}}^{*}$, $\varepsilon_{F}$ is the Fermi-energy, $\theta(x)$ is Heaviside function. The photoemission spectral distribution displays replica peaks with interval $\omega_{0}=150 \mathrm{meV}$. And the spectral weights roughly follow a Poisson distribution with factor $p=\sum_{\bar{\boldsymbol{q}}} \frac{N_{v}\left| g_{\bar{\boldsymbol{q}}} \right|^{2}}{N_{m}\omega_{0}^{2}}$ .The numerical data by the frozen-phonon method indicate that $p=0.11$. Including electronic interactions (much smaller than $\omega_{0}$) would change the single-particle Fermi distribution in Eq. (31) into complicated many-body distribution but does not affect the relative spectral weights among replicas.

Though Eq. (31) is the zero-temperature result, its extension to finite temperatures requires only an ensemble summation of the initial state to replace the electronic ground state $|\left. \psi_{G} \right\rangle$. Considering the typical value of the phonon frequency $\omega_{0}$ is far above room temperature, the initial phonon state can always be regarded as the vacuum. Therefore, the spectral distribution among all replica peaks remains the Poisson distribution in Eq. (31) for finite temperature, with the only difference stemming from the redistribution within each replica, which reflects the thermal flat-band electronic states. In the non-interacting limit, the finite-temperature results can be calculated via substituting $\theta\left[ \varepsilon_{F}-\varepsilon_{\alpha}^{*}(\bar{\boldsymbol{k}^{\boldsymbol{'}}}) \right]$ by Fermi-Dirac distribution $n_{F}\left[ \varepsilon_{\alpha}^{*}(\bar{\boldsymbol{k}^{\boldsymbol{'}}})-\varepsilon_{F} \right]$ in Eq. (31).


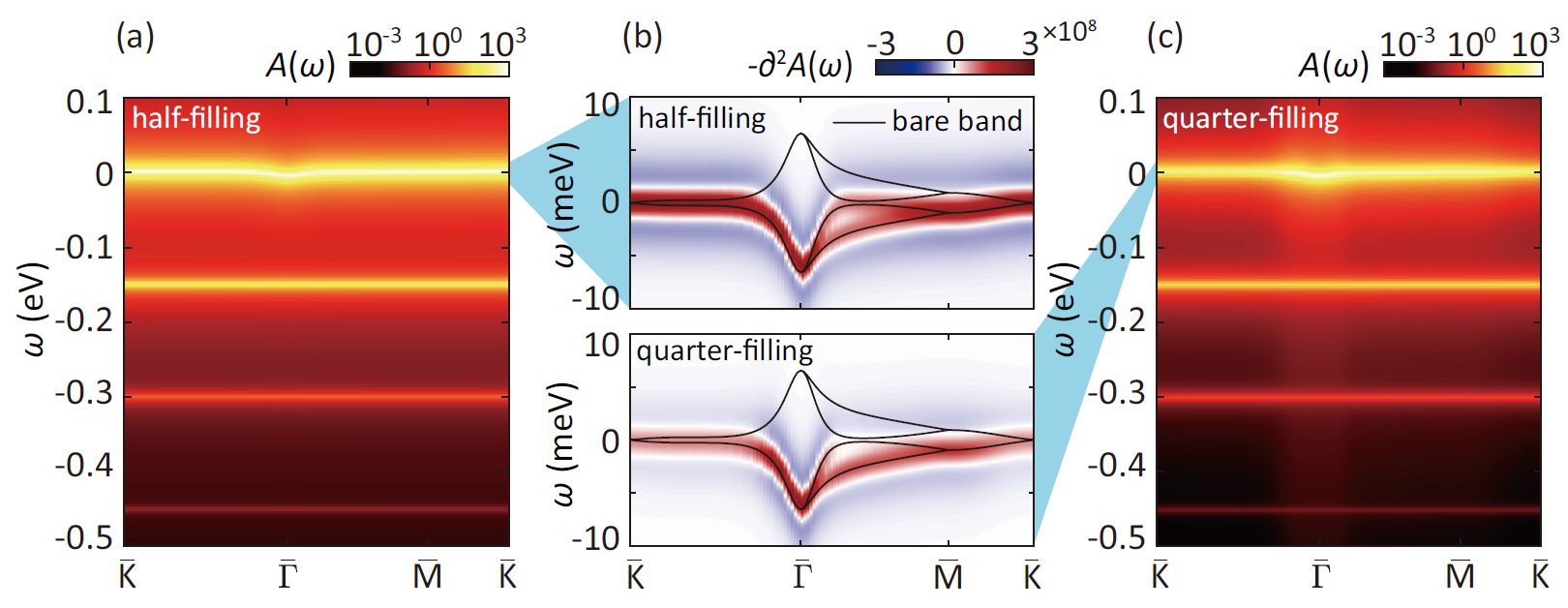


**Fig. S12 | Simulated photoemission spectra.** The photoemission spectra at (a) half-filling and (c) quarter-filling. The Upper (lower) panel of (b) shows the zoomed-in second-derivative spectrum of the zeroth replica at half-filling (quarter-filling), where the solid lines are the bare bands without EPC. All figures are calculated at $\omega_{0}=150 meV$, $T=5 K$. $N_{v}=120$ and $\Gamma=2.5 meV$.

**X. Effect of nearly aligned hBN on TBG**

To analyse the impact of aligned hBN substrate, we consider the electron-phonon model of TBG electrons and treat hBN as a background potential. Similar to the frozen phonon model in Eq. (17), we employ the continuum model (extended BM model) of the bare TBG and restrict the discussion of this section to only one branch of the $\bar{\boldsymbol{q}}\boldsymbol{=}\bar{\boldsymbol{0}}$ phonon for simplicity. The Hamiltonian reads

$$H_{TBG/hBN}= \sum_{\bar{\boldsymbol{k}}\sigma} \boldsymbol{c}_{\bar{\boldsymbol{k}}\sigma}^{\left( \mathrm{TAPW} \right)\dagger}h_{\bar{\boldsymbol{k}}}\boldsymbol{c}_{\bar{\boldsymbol{k}}\sigma}^{\left( \mathrm{TAPW} \right)}-\frac{1}{\sqrt{N_{m}}}\sum_{\bar{\boldsymbol{k}}\sigma} \boldsymbol{c}_{\bar{\boldsymbol{k}}\sigma}^{\left( \mathrm{TAPW} \right)\dagger}M_{\bar{\boldsymbol{0}}}\boldsymbol{c}_{\bar{\boldsymbol{k}}\sigma}^{\left( \mathrm{TAPW} \right)}\left( a_{\bar{\boldsymbol{0}}}+a_{\bar{\boldsymbol{0}}}^{\dagger} \right)+\omega_{0}a_{\bar{\boldsymbol{0}}}^{\dagger}a_{\bar{\boldsymbol{0}}}+\sum_{\bar{\boldsymbol{k}}\sigma} \boldsymbol{c}_{\bar{\boldsymbol{k}}\sigma}^{\left( \mathrm{TAPW} \right)\dagger}V_{\mathrm{hBN}}\boldsymbol{c}_{\bar{\boldsymbol{k}}\sigma}^{\left( \mathrm{TAPW} \right)}$$

*(32)*

where the potential arises from assigned hBN substrate is

$\left[ V_{\mathrm{hBN}} \right]=V\delta_{\boldsymbol{G}\boldsymbol{G}^{\boldsymbol{'}}}\delta_{\eta\eta^{'}}\delta_{l,\mathrm{bottom}}\sigma_{\alpha,\alpha^{'}}^{z}$ *(33)*

Here, $(\eta,l,\sigma,\alpha)$ label the valley, layer, spin and A/B site, respectively. $V_{\mathrm{hBN}}$ can be regarded as the mass term of the bottom-layer graphene, resulting from the broken space-inversion (P) symmetry by the aligned hBN substrate. *Ab initio* calculations of monolayer graphene/hBN with full lattice relaxation yield the estimate $V=3.62 meV$^29^, but experiments suggest that $V$ is significantly larger. A rough estimate is obtained from experiments on monolayer graphene nearly aligned with hBN ^30^, which shows that the band gap at the neutrality point is around 35 meV, implying $V\approx17 \mathrm{meV}$. We adopt $V=17 \mathrm{meV}$ in the following calculation. Without consider the structure relaxation, we employ the same EPC matrix $M_{0}$ as Eq. (17).

We generalize the effective flat-band model of Eq. (18) by projecting the TBG/hBN continuum model in the form of Eq. (32) onto the flat-band manifold, which diagonalizes the electron kinetic terms $P_{\bar{\boldsymbol{k}}}^{\dagger}(h_{\bar{\boldsymbol{k}}}+V_{\mathrm{hBN}})P_{\bar{\boldsymbol{k}}}$ instead. With the generalized projection operator $P_{\bar{\boldsymbol{k}}}$ and flat-band energies $\left\{ \varepsilon_{\bar{\boldsymbol{k}}} \right\}$ , the EPC matrix $M_{0}$ of the continuum model can also be projected to the flat-band manifold as

$P_{\bar{\boldsymbol{k}}}^{\dagger}M_{\bar{\boldsymbol{0}}}P_{\bar{\boldsymbol{k}}}=\left( \begin{matrix} & m_{\bar{\boldsymbol{k}}} \\ m_{\bar{\boldsymbol{k}}}^{\dagger} & \end{matrix} \right)$ *(34)*

where $m_{\bar{\boldsymbol{k}}}$ is an off-diagonal 2 × 2 submatrix that scatters the electron from valley *K′* to *K* due to the cross-valley nature of $M_{0}$ (see Sec. VIII). With aligned hBN substrate, the projected matrix $m_{\bar{\boldsymbol{k}}}$ differs since the flat-band wave functions in TBG discussed in Sec. IX. For convenience, we consider the SVD decomposition of $m_{\bar{\boldsymbol{k}}}$,

$m_{\bar{\boldsymbol{k}}}=V_{\bar{\boldsymbol{k}}}\left( \begin{matrix} g_{\bar{k_{1}}} & \\ & g_{\bar{k_{2}}} \end{matrix} \right)W_{\bar{\boldsymbol{k}}}^{\dagger}$ *(35)*

where $V_{\bar{\boldsymbol{k}}}$ and $W_{\bar{\boldsymbol{k}}}$ are both 2 × 2 unitary matrices. Here, the singular values remain almost momentum-independent and degenerate, i.e. $g_{\bar{k_{1}}}=g_{\bar{k_{2}}}\equiv g_{\bar{\boldsymbol{k}}}$ with aligned hBN substrate. That being said, $m_{\bar{\boldsymbol{k}}}$ can be further simplified to$m_{\bar{\boldsymbol{k}}}=g_{\bar{\boldsymbol{k}}}(V_{\bar{\boldsymbol{k}}}W_{\bar{\boldsymbol{k}}}^{\dagger})$. More importantly, these singular values are almost identical for TBG and TBG/hBN, though with different projectors $P_{\bar{\boldsymbol{k}}}$. These observations indicate that rotating the flat-band basis to the singular vectors of the EPC matrix is more instructive in discussing the impact of hBN.

Therefore, we consider the following unitary transformation to rotate the flat-bands,

$U_{\bar{\boldsymbol{k}}}=\left( \begin{matrix} \sigma_{0} & \\ & W_{\bar{\boldsymbol{k}}}V_{\bar{\boldsymbol{k}}}^{\dagger} \end{matrix} \right)$ *(36)*

where $\sigma_{0}$ denotes the 2 × 2 identity. In this rotated basis, the EPC matrix can be simplified to

$U_{\bar{\boldsymbol{k}}}^{\dagger}P_{\bar{\boldsymbol{k}}}^{\dagger}M_{\bar{\boldsymbol{0}}}P_{\bar{\boldsymbol{k}}}U_{\bar{\boldsymbol{k}}}=\left( \begin{matrix} & {g_{\bar{\boldsymbol{k}}}\sigma}_{0} \\ {g_{\bar{\boldsymbol{k}}}\sigma}_{0} & \end{matrix} \right)$ *(37)*

Note that one can choose to keep the *K*-valley component of $P_{\bar{\boldsymbol{k}}}^{\dagger}(h_{\bar{\boldsymbol{k}}}+V_{\mathrm{hBN}})P_{\bar{\boldsymbol{k}}}$ diagonalized using Eq. (36). Due to the lack of inversion symmetry with aligned hBN, one cannot keep the components in both valleys diagonalized simultaneously, which is the primary difference from the TBG model in Eq. (18). After this basis transformation, the EPC matrix becomes identical for TBG with and without aligned hBN. However, when $P_{\bar{\boldsymbol{k}}}^{\dagger}(h_{\bar{\boldsymbol{k}}}+V_{\mathrm{hBN}})P_{\bar{\boldsymbol{k}}}$cannot be simultaneously diagonalized on both valleys, they mix the eigenstates of the electronic Hamiltonian. That being said, different from the simple form of Eq. (18) for TBG-only systems, the hBN-aligned TBG flat-band Hamiltonian becomes

$$H_{\mathrm{flat}}= \sum_{\bar{\boldsymbol{k}}\eta\sigma} \boldsymbol{c}_{\bar{\boldsymbol{k}}\eta\sigma}^{\left( \mathrm{flat} \right)\dagger}(h_{\bar{\boldsymbol{k}}\eta0}\sigma_{0}+\boldsymbol{h}_{\bar{\boldsymbol{k}}\eta}\boldsymbol{\cdot\sigma})\boldsymbol{c}_{\bar{\boldsymbol{k}}\eta\sigma}^{\left( \mathrm{flat} \right)}-\frac{1}{\sqrt{N_{m}}}\sum_{\bar{\boldsymbol{k}}\eta\sigma} g_{\bar{\boldsymbol{k}}}\boldsymbol{c}_{\bar{\boldsymbol{k}}\bar{\eta}\sigma}^{\left( \mathrm{flat} \right)\dagger}\sigma_{0}\boldsymbol{c}_{\bar{\boldsymbol{k}}\eta\sigma}^{\left( \mathrm{flat} \right)}\left( a_{\bar{\boldsymbol{0}}}+a_{\bar{\boldsymbol{0}}}^{\dagger} \right)+\omega_{0}a_{\bar{\boldsymbol{0}}}^{\dagger}a_{\bar{\boldsymbol{0}}}$$

*(38)*

where $\bar{\eta}$ denotes the opposite valley of $\eta$. For TBG without aligned hBN, the kinetic matrix, i.e. $h_{\bar{\boldsymbol{k}}\eta0}\sigma_{0}+\boldsymbol{h}_{\bar{\boldsymbol{k}}\eta}\boldsymbol{\cdot\sigma}$ in Eq. (38), reduces to a diagonal matrix $h_{\bar{\boldsymbol{k}}\eta0}\sigma_{0}+h_{\bar{\boldsymbol{k}}\eta z}\sigma_{z}$, indicating the preserved electronic eigen basis after the transformation. In contrast, off-diagonal terms occur for TBG with aligned hBN, leading to non-commuting relation with the EPC matrix even in the flat-band limit. As we will demonstrate below, this property strongly affects the polaronic dressing.

Analytically solving the hBN-aligned flat-band Hamiltonian in Eq. (38) is generally impractical. To reflect the trend of replicas observed in our experiments, we numerically analyse the polaronic dressing using the variational non-Gaussian transformation. We follow the assumptions justified in Sec. IX for the TBG EPC strengths and drop the momentum dependence of EPC as $g_{\bar{\boldsymbol{q}}}\approx g$. For the convenience of polaronic transformation, we again change to an EPC-diagonal basis by $\boldsymbol{d}_{\bar{\boldsymbol{k}}\sigma}=Q^{\dagger}\boldsymbol{c}_{\bar{\boldsymbol{k}}\sigma}^{(\mathrm{flat})}$ with

$Q=\frac{1}{\sqrt{2}}\left( \begin{matrix} 1 & 0 & 1 & 0 \\ 0 & 1 & 0 & 1 \\ 1 & 0 & -1 & 0 \\ 0 & 1 & 0 & -1 \end{matrix} \right)$ *(39)*

such that EPC matrix $Q^{\dagger}\left( \begin{matrix} & g\sigma_{0} \\ g\sigma_{0} & \end{matrix} \right)Q=g\eta$, where $\eta=\mathrm{diag}(1,1,-1,-1)$. In this basis, we formally recovered the flat-band EPC model in Eq. (18), with the kinetic matrix replaced by

$\tilde{h}(\bar{\boldsymbol{k}})=Q^{\dagger}\left( \begin{matrix} h_{\bar{\boldsymbol{k}}K0}\sigma_{0}+\boldsymbol{h}_{\bar{\boldsymbol{k}}K}\boldsymbol{\cdot\sigma} & \\ & h_{\bar{\boldsymbol{k}}K^{'}0}\sigma_{0}+\boldsymbol{h}_{\bar{\boldsymbol{k}}K^{'}}\boldsymbol{\cdot\sigma} \end{matrix} \right)Q$ . *(40)*

Then similar to the procedure of Sec. IX, we employ a non-Gaussian transformation to decoupling the electron-phonon term. Different from the Lang-Firsov transformation with fixed exponential factors, here we generalize this transformation into a variational one^31,32^. Since the EPC matrix and kinetic matrix, on the four-band basis, do not commute with each other, the decoupling process induces polaronic dressing back to the kinetic energy, and, therefore, the latter competes with the EPC term. Such competition is already reflected in Eq. (23) of the TBG system. Although the electronic bandwidth of flat-band systems is negligible compared to the EPC strength and phonon energy, the hBN-induced potential is substantial, leading to a non-commuting $\tilde{h}(\bar{\boldsymbol{k}})$ with the EPC matrix. In order to balance these two competing dressing processes, we adopted a variational ansatz of the ground state $|\left. \Psi_{G} \right\rangle=U_{\mathrm{NGS}}^{\dagger}\left( \lambda\right)|\left. \psi_{e} \right\rangle|\left. 0_{\mathrm{ph}} \right\rangle$ with^31,32^

$U_{\mathrm{NGS}}^{\dagger}\left( \lambda\right)=exp\left[ \frac{\lambda}{\sqrt{N_{m}}}\sum_{\bar{\boldsymbol{R}}\sigma\bar{\boldsymbol{q}}\nu} e^{-i\bar{\boldsymbol{R}}\cdot\bar{\boldsymbol{q}}}\boldsymbol{d}_{\bar{\boldsymbol{R}}\sigma}^{\dagger}\eta\boldsymbol{d}_{\bar{\boldsymbol{R}}\sigma}\left( a_{\bar{\boldsymbol{q}}\nu}-a_{-\bar{\boldsymbol{q}}\nu}^{\dagger} \right) \right]$ *(41)*

where $\lambda$ is the variational parameter in contrast to the fixed $g/\omega_{0}$ of the Lang-Firsov transformation in Sec. IX. Similar to Eq. (24) and (30), the total energy for this ground-state ansatz becomes

$$E_{\mathrm{tot}}\left( \lambda\right)=\left\langle0_{\mathrm{ph}} \right.\left| \left\langle\psi_{e} | U_{\mathrm{NGS}}\left( \lambda\right)HU_{\mathrm{NGS}}^{\dagger}\left( \lambda\right) | \psi_{e} \right\rangle\right|\left. 0_{\mathrm{ph}} \right\rangle$$

$=E_{\mathrm{kin}}-\frac{N_{v}}{N_{m}}(2g\lambda-\omega_{0}\lambda^{2})\sum_{\bar{\boldsymbol{R}}\bar{\boldsymbol{R}^{\boldsymbol{'}}}\sigma\sigma^{'}\bar{\boldsymbol{q}}} e^{i\left( \bar{\boldsymbol{R}^{\boldsymbol{'}}}\boldsymbol{-}\bar{\boldsymbol{R}} \right)\cdot\bar{\boldsymbol{q}}}\left\langle\psi_{e} | \left( \boldsymbol{d}_{\bar{\boldsymbol{R}}\sigma}^{\dagger}\eta\boldsymbol{d}_{\bar{\boldsymbol{R}}\sigma} \right)\left( \boldsymbol{d}_{\bar{\boldsymbol{R}^{\boldsymbol{'}}}\sigma^{'}}^{\dagger}\eta\boldsymbol{d}_{\bar{\boldsymbol{R}^{\boldsymbol{'}}}\sigma^{'}} \right) | \psi_{e} \right\rangle$ *(42)*

with the normalized kinetic energy

$E_{\mathrm{kin}}=\sum_{\bar{\boldsymbol{R}}\bar{\boldsymbol{R}^{\boldsymbol{'}}}\sigma} \sum_{\alpha\alpha'} \exp\left\{ -\frac{N_{v}}{N_{m}}\sum_{\bar{\boldsymbol{q}}} \lambda^{2}\left[ 1-\eta_{\alpha\alpha}\eta_{\alpha'\alpha'}\cos\bar{\boldsymbol{q}}\cdot\left( \bar{\boldsymbol{R}}\boldsymbol{-}\bar{\boldsymbol{R}^{\boldsymbol{'}}} \right) \right] \right\}\tilde{h}_{\bar{\boldsymbol{R}}\bar{\boldsymbol{R}^{\boldsymbol{'}}},\alpha\alpha'}\left\langle\psi_{e} | \boldsymbol{d}_{\bar{\boldsymbol{R}}\alpha\sigma}^{\dagger}\boldsymbol{d}_{\bar{\boldsymbol{R}^{\boldsymbol{'}}}\alpha'\sigma} | \psi_{e} \right\rangle$ *(43)*

Here, $\alpha$ and $\alpha'$ label the four-component (rotated) band indices. The value of $\lambda$ is obtained by minimizing the total energy as $\partial E_{\mathrm{tot}}\left( \lambda\right)/\partial\lambda=0$. Note that in the flab-band TBG limit, where $E_{\mathrm{kin}}\approx0$, there is a trivial solution $\lambda=g/\omega_{0}$ by minimizing the second term, which automatically returns to the Lang-Firsov transformation.

In the TBG with aligned hBN, there is no trivial solution since the hBN-induced shift in site energy [i.e., $\boldsymbol{h}_{\bar{\boldsymbol{k}}K^{'}}$ in Eq. (40)] cannot be ignored. To qualitatively discuss the impact of hBN and solve the variational problem analytically, we first assume the flat-band limit and leave the bandwidth discussion to Sec. XI. Thus, all spatial hoppings are ignored, and the only contributions to the kinetic terms come from site energies affected by hBN

$\tilde{h}\left( \bar{\boldsymbol{k}} \right)=\tilde{h}=Q^{\dagger}\left( \begin{matrix} h_{K} & \\ & h_{K^{'}} \end{matrix} \right)Q$ *(44)*

In this simplified scenario, we obtain the analytical solution for $\min E_{\mathrm{tot}}\left( \lambda\right)$, which is reached at

$\lambda=\frac{{gE}_{\mathrm{int}}}{\omega_{0}E_{\mathrm{int}}-e^{-2N_{v}\lambda^{2}}{\Delta E}_{\mathrm{kin}}}$ *(45)*

Here, the two key control factors are the valley-differential kinetic energy, which is simplified in the flatband basis as

${\Delta E}_{\mathrm{kin}}=\frac{1}{N_{m}}\sum_{\bar{\boldsymbol{k}}\sigma} \left\langle\psi_{e} | \boldsymbol{c}_{\bar{\boldsymbol{k}}\sigma}^{(\mathrm{flat})\dagger}\left( \begin{matrix} h_{K}-h_{K'} & \\ & h_{K'}-h_{K} \end{matrix} \right)\boldsymbol{c}_{\bar{\boldsymbol{k}}\sigma}^{(\mathrm{flat})} | \psi_{e} \right\rangle$ *(46)*

and the phonon-mediated local interaction

$E_{\mathrm{int}}=\frac{1}{N_{m}}\sum_{\bar{\boldsymbol{R}}} \left\langle\psi_{e} | \left[ \sum_{\sigma} \boldsymbol{c}_{\bar{\boldsymbol{R}}\sigma}^{\left( \mathrm{flat} \right)\dagger}\left( \begin{matrix} & \sigma_{0} \\ \sigma_{0} & \end{matrix} \right)\boldsymbol{c}_{\bar{\boldsymbol{R}}\sigma}^{\left( \mathrm{flat} \right)} \right]^{2} | \psi_{e} \right\rangle$ *(47)*

It is worth noting that the original electronic interaction is not included in our derivation. If the multi-band Coulomb interaction commutes with the non-Gaussian transformation in Eq. (46), it does not affect the variation of $\lambda$. In contrast, if it includes interactions non-commuting with the transformation in Eq. (46), the generated terms will enter the $E_{\mathrm{int}}$ and correct the dressing factor. While the impact of Coulomb interactions is beyond the scope of this paper, the presence of local Coulomb interactions is qualitatively believed to help the formation of polarons^33^.

To give a qualitative picture of the hBN-suppressed polaronic dressing, we make a simple assumption about the electronic ground state in Eq. (47) as the half-filling Fermi-sea. Then, with the TBG flat-band parameters, we can evaluate the two factors in Eq. (45), which is $E_{\mathrm{int}}=2$ and ${\Delta E}_{\mathrm{kin}}\approx0$, then $\lambda_{\mathrm{TBG}}=g/\omega_{0}$ and we reproduce the conclusion and spectral distribution in Sec. IX; in contrast, for hBN-aligned TBG, we approximate the potential as $h_{K}=\Delta\sigma_{z}$ and $h_{K^{'}}=-\Delta\sigma_{z}$, resulting in ${\Delta E}_{\mathrm{kin}}=-4\Delta$ and $E_{\mathrm{int}}=2$. Then $\lambda_{TBG/hBN}$ can be self-consistently determined by

$\lambda_{TBG/hBN}=\frac{g}{\omega_{0}+2\Delta e^{-2N_{v}{\lambda_{TBG/hBN}}^{2}}}$ *(48)*

While simulating the excited-state spectrum in this generalized model and variational ansatz is complicated, we can follow the conclusion in Sec. IX and estimate the relative replica intensity as $p=N_{v}\lambda^{2}$. Thus, the phonon-induced replica is naturally suppressed by the aligned hBN potential, as shown in Fig. 4 of the main text.

**XI. Impact of twist angle and bandwidth**

In the discussion of the hBN substrate effect, we have focused on the valley splitting in Eq. (44) and ignored the bandwidth of each flatband. In reality, the bandwidth is nonzero and is sensitive to the twist angle in TBG. Therefore, we generalize the analysis into finite bandwidth in this section, reflecting the qualitative impact of twist angle on the polaronic dressing.

Similar to the valley splitting, any finite momentum dependence of $\tilde{h}\left( \bar{\boldsymbol{k}} \right)$ leads to additional terms non-commuting with the non-Gaussian transformation $U_{\mathrm{NGS}}^{\dagger}\left( \lambda\right)$. In this case, we should take into account the nonlocal hopping terms $\tilde{h}_{\bar{\boldsymbol{R}}{\bar{\boldsymbol{R}}}^{'},\alpha\alpha^{'}}$ in Eq. (43), which are the Fourier transform of the momentum-dependent $\tilde{h}\left( \bar{\boldsymbol{k}} \right)$. Therefore, the suddle-point solution for variational dressing factor is generalized into

$\lambda=\frac{{gE}_{\mathrm{int}}}{\omega_{0}E_{\mathrm{int}}-e^{-2N_{v}\lambda^{2}}{\Delta E}_{\mathrm{kin}}-e^{-N_{v}\lambda^{2}}\bar{E}_{\mathrm{disp}}}$ *(49)*

The dispersive part of kinetic energy reflects the band structure is

$\bar{E}_{\mathrm{disp}}=\frac{1}{N_{m}}\sum_{\bar{\boldsymbol{k}}\sigma} \left\langle\psi_{e} | \boldsymbol{c}_{\bar{\boldsymbol{k}}\sigma}^{(\mathrm{flat})\dagger}\left( \begin{matrix} h_{K}\left( \bar{\boldsymbol{k}} \right)-\left\langle h_{K^{'}} \right\rangle& \\ & h_{K^{'}}\left( \bar{\boldsymbol{k}} \right)-\left\langle h_{K^{'}} \right\rangle\end{matrix} \right)\boldsymbol{c}_{\bar{\boldsymbol{k}}\sigma}^{(\mathrm{flat})} | \psi_{e} \right\rangle$ *(50)*

Based on the intuition obtained in Sec. IX, the finite bandwidth, contributes additional kinetic energy to the competition with EPC. The consequence is reflected in the denominator of Eq. (49), where the bandwidth effect further suppresses the polaronic dressing and pushes $\lambda$ farther from the Lang-Firsov limit ($g/\omega_{0}$).

To demonstrate such a suppression, we mimic the $\tilde{h}\left( \bar{\boldsymbol{k}} \right)$ by a honeycomb nearest-neighbor tight-binding model with valley degrees of freedom. For the qualitative demonstration, we do not consider the complicated flatband structure for any specific twist angle. Instead, we use this toy model, with basic lattice and valley symmetries, to show the impact of both hBN potential $\Delta$ and bandwidth $w$. In the momentum space, the hopping reads

$h_{K}\left( \bar{\boldsymbol{k}} \right)=\left( \begin{matrix} \Delta& -tf(\bar{\boldsymbol{k}}) & & \\ -tf^{*}(\bar{\boldsymbol{k}}) & -\Delta& & \\ & & -\Delta& -tf^{*}(\bar{\boldsymbol{k}}) \\ & & -tf(\bar{\boldsymbol{k}}) & \Delta\end{matrix} \right)$ *(51)*

where $f\left( \bar{\boldsymbol{k}} \right)=\sum_{j=1}^{3} e^{i\bar{\boldsymbol{k}}\boldsymbol{\cdot}{\bar{\boldsymbol{\delta}}}_{j}}$ with ${\bar{\boldsymbol{\delta}}}_{j}$the vectors connecting nearest-neighbor sites. For given bandwidth $w$, $t=\sqrt{w^{2}+2w\Delta}/3$ We assume the electron ground state is the half-filling Fermi-sea state determined by the bare electron kinetic term. One finds

$E_{\mathrm{int}}=2+2\left( \frac{1}{N_{m}}\sum_{\bar{\boldsymbol{k}}} \frac{\Delta}{\sqrt{\Delta^{2}+\left| tf(\bar{\boldsymbol{k}}) \right|^{2}}} \right)^{2}$ *(52)*

${\Delta E}_{\mathrm{kin}}=-\frac{8}{N_{m}}\sum_{\bar{\boldsymbol{k}}} \frac{\Delta^{2}}{\sqrt{\Delta^{2}+\left| tf(\bar{\boldsymbol{k}}) \right|^{2}}}$ *(53)*

$E_{\mathrm{disp}}=-\frac{4}{N_{m}}\sum_{\bar{\boldsymbol{k}}} \frac{\left| tf(\bar{\boldsymbol{k}}) \right|^{2}}{\sqrt{\Delta^{2}+\left| tf(\bar{\boldsymbol{k}}) \right|^{2}}}$ *(54)*

Then λ can be self-consistently determined by Eq. (52) and gives the corresponding Poisson factor of band replica $p=N_{v}\lambda^{2}$ as a function of $\Delta$ and bandwidth $w$ in Fig. 4 of the main text. Note that ${\Delta E}_{\mathrm{kin}}$ and $E_{\mathrm{disp}}$ (both negative) contribute to the suppression of $\lambda$. ${\Delta E}_{\mathrm{kin}}$ term arises from the valley band-inversion induced by the aligned hBN substrate, and vanishes for bare TBG. $\left| E_{\mathrm{disp}} \right|$ is positively correlated to the bandwidth and is dominant in the large-twisted-angle limit of TBG.

**XII Exact diagonalization simulation in the chiral limit**


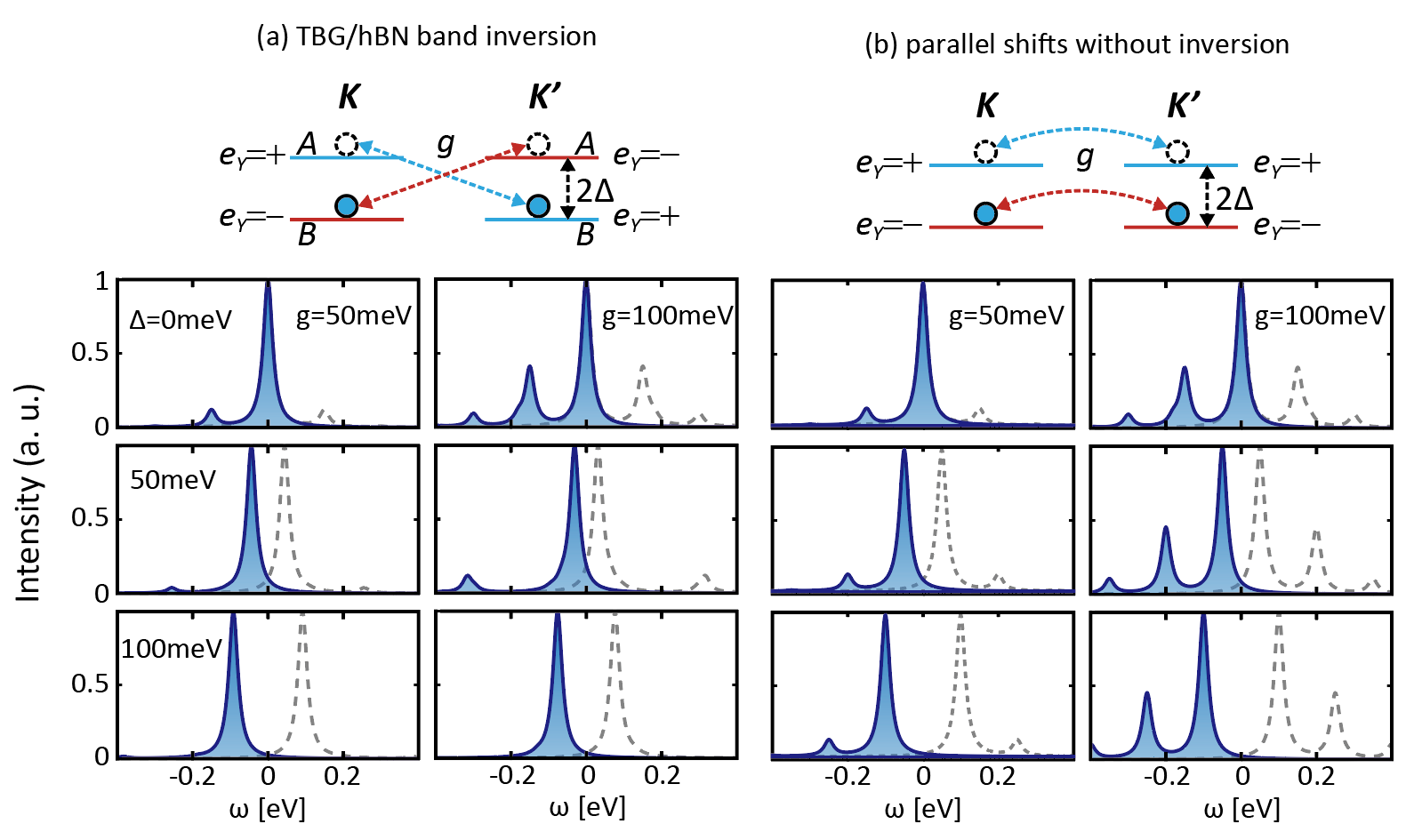


**Fig. S13 | Influence of the hBN alignment on the flat band replicas.** (a) Upper: Schematic of the band inversion induced by the hBN substrate potential Δ. $e_{Y}$ labels the Chern-basis index, whose relative energies are inverted between *K* and *K*’ valleys for aligned TBG/hBN. Lower: Spectral function obtained for different hBN potentials under coupling strength $g=50$ meV (left) and 100 meV (right). The electron removal parts of the spectral function are indicated by filled shades. In the two-site toy model simualtion, we set the phonon energy $\omega_{0}=150 \mathrm{meV}$, and temperature $T=$116 K. A lifetime broadening$\Gamma=15 \mathrm{me}$V is added. (b) Similar model with (a) but if the potential energy does not invert the two bands at the *K* and *K*’ valleys. The phonon-induced replicas persist at large Δ.

The discussions in Sec. IX-XI are based on the variational non-Gaussian method, with an *a priori* assumption for the wavefunction. Although this assumption has been benchmarked with exact solutions for the Holstein-type phonon^34^, its accuracy in cross-valley phonons and multi-band electronic systems has not been numerically benchmarked. Therefore, for the completeness of this work, we further apply a numerically exact method (exact diagonalization, or ED) to solve a toy model in the chiral limit, representing the TBG system discussed in this paper. Since ED enumerates the entire Hilbert space, it is restricted to a very small cluster.

In the chiral limit, the EPC of the flat bands in TBG has a simplified form, which will be discussed below. We rewrite the electronic structure into the Chern basis, which relates to the eigen-basis by

$\gamma_{\bar{\boldsymbol{k}}\boldsymbol{,}\eta,e_{Y},\sigma}^{\dagger}=\frac{1}{\sqrt{2}}\left( c_{\bar{\boldsymbol{k}},\eta,\alpha=1,\sigma}^{\left( \mathrm{flat} \right)\dagger}+ie_{Y}c_{\bar{\boldsymbol{k}},\eta,\alpha=2,\sigma}^{\left( \mathrm{flat} \right)\dagger} \right),$ *(55)*

where $\eta$ is the valley index, $\sigma$ is the spin index, $\alpha=1,2$ labels two eigenstates of flat bands for TBG (per valley per spin), $e_{Y}=\pm$ is the index labeling the Chern-basis. The corresponding eigen-wavefunctions in the Chern basis carry the Chern number $e_{Y}$ in the moiré Brillouin zone, but they are not the eigenstates of the TBG Hamiltonian. On the Chern basis, the single-particle effective Hamiltonian for the flat bands has the form^27^

$\tilde{H}_{e}=\sum_{\bar{\boldsymbol{k}}\eta s,e_{Y}e_{Y}^{'}} \gamma_{\bar{\boldsymbol{k}},\eta,e_{Y},\sigma}^{\dagger}\left[ d_{0,\eta}\left( \bar{\boldsymbol{k}} \right)\delta_{e_{Y},e_{Y}^{'}}+d_{x,\eta}\left( \bar{\boldsymbol{k}} \right)(1-\delta_{e_{Y},e_{Y}^{'}}) \right]\gamma_{\bar{\boldsymbol{k}},\eta,e_{Y}^{'},\sigma}$, *(56)*

where the diagonal and off-diagonal kinetic matrix elements are

$d_{0,\eta}\left( \bar{\boldsymbol{k}} \right)=\frac{1}{2}\left( \varepsilon_{\bar{\boldsymbol{k}},\eta,1}+\varepsilon_{\bar{\boldsymbol{k}},\eta,2} \right), d_{x,\eta}\left( \bar{\boldsymbol{k}} \right)=\frac{1}{2}\left( \varepsilon_{\bar{\boldsymbol{k}},\eta,1}-\varepsilon_{\bar{\boldsymbol{k}},\eta,2} \right)$ , *(57)*

and $\varepsilon_{\bar{\boldsymbol{k}},\eta,\alpha=1,2}$ are the eigen-energies of two flat bands that are created by the operator $c_{\bar{\boldsymbol{k}},\eta,\alpha=1,2,\sigma}^{\left( \mathrm{flat} \right)\dagger}$ in TBG. As both $d_{0,\eta}\left( \bar{\boldsymbol{k}} \right)$ and $d_{x,\eta}\left( \bar{\boldsymbol{k}} \right)$ are real, time reversal symmetry requires $\tilde{h}_{-\bar{\boldsymbol{k}}\boldsymbol{,-}\eta}=\tilde{h}_{\bar{\boldsymbol{k}}\boldsymbol{,}\eta}$. The chiral symmetry requires $d_{0,\eta}\left( \bar{\boldsymbol{k}} \right)=0$. In the chiral limit, the Chern-basis is fully sub-lattice polarized, and one can derive the connection between the Chern basis index and sub-lattice index $a$ by $e_{Y}=-\eta\left( -1 \right)^{a}$, where $\eta=+, -$ for $K, K'$, and $a=1,2$ for the $A,B$ sub-lattice. As the electron-iTO-phonon interaction couples the electrons at the sublattice $A$ and the valley $\eta$ to the electrons at the sublattice $B$ and the valley $\bar{\eta}=-\eta$, this interaction is diagonal on the Chern-basis in the chiral limit, which has been proved mathematically in Ref. 27. Thus, the electron-iTO-phonon interaction has the form

$\tilde{H}_{e-ph}=\frac{1}{\sqrt{N_{m}}}\sum_{\bar{\boldsymbol{k,}}\eta,e_{Y},\sigma} \tilde{g}_{\bar{\boldsymbol{k}},\eta,e_{Y}}\gamma_{\bar{\boldsymbol{k}},\eta,e_{Y},\sigma}^{\dagger}\gamma_{\bar{\boldsymbol{k}},\bar{\eta},e_{Y},\sigma}\left( a_{\bar{\boldsymbol{0}}}+a_{\bar{\boldsymbol{0}}}^{\dagger} \right)$, *(58)*

within the same approximation used for Eq. (32) and (38) in Sec. X and in the chiral limit. The hermicity condition requires $\tilde{g}_{\bar{\boldsymbol{k}},\eta,e_{Y}}^{*}=\tilde{g}_{\bar{\boldsymbol{k}},-\eta,e_{Y}}$ and time-reversal symmetry requires $\tilde{g}_{\bar{\boldsymbol{k}},\eta,e_{Y}}^{*}=\tilde{g}_{-\bar{\boldsymbol{k}},-\eta,-e_{Y}}$.

To conduct exact diagonalization (ED) in a small system, we further assume $\tilde{g}_{\bar{\boldsymbol{k}},\eta,e_{Y}}$ independent of $\bar{\boldsymbol{k}}$ here. Therefore, the EPC matrix is reduced to only one independent complex value for $\tilde{g}_{\eta=\boldsymbol{K,}e_{Y}=+}=g$, and from the hermicity condition and time-reversal symmetry, one can show $\tilde{g}_{K^{'},-}=\tilde{g}_{K^{'},+}=g^{*}$ and $\tilde{g}_{K,-}=g$ Thus, we have

$\tilde{H}_{e-ph}=\frac{1}{\sqrt{N_{m}}}\sum_{\bar{\boldsymbol{k}}e_{Y}\sigma} \left( g\gamma_{\bar{\boldsymbol{k}},K,e_{Y},\sigma}^{\dagger}\gamma_{\bar{\boldsymbol{k}},K^{'},e_{Y},\sigma}+h.c. \right)\left( a_{\bar{\boldsymbol{0}}}+a_{\bar{\boldsymbol{0}}}^{\dagger} \right).$ *(59)*

The hBN potential describes a staggered potential on sublattices, namely a potential $\Delta$ on the $A$ site and $-\Delta$ on the $B$ site, as discussed in Sec. X. From the connection between the Chern basis index and the sub-lattice index, $\left( -1 \right)^{a}={-e}_{Y}\eta$ , one can project the hBN potential onto the Chern basis

$\tilde{H}_{\mathrm{hBN}}=\Delta\sum_{\bar{\boldsymbol{k}}\eta\sigma e_{Y}} e_{Y}\eta\gamma_{\bar{\boldsymbol{k}},\eta,e_{Y},\sigma}^{\dagger}\gamma_{\bar{\boldsymbol{k}},\eta,e_{Y},\sigma}$ *(60)*

The full Hamiltonian $\tilde{H}_{\mathrm{eff}}=\tilde{H}_{\bar{\boldsymbol{k}}}^{(e)}+\tilde{H}_{\mathrm{hBN}}+\tilde{H}_{e-ph}$ shares a similar form as Eq. (51) in Sec. XI. Fig. S13 (a) illustrates the general picture of the flat bands under the hBN potential $\Delta$ and the electron-iTO-phonon interaction $g$.

This flat-band model in the chiral limit separates all fermionic momentum indices. Therefore, we simplify the problem into a four-orbital model that captures the behaviour of a single $\bar{\boldsymbol{k}}$ (for both K and K’):

$$\tilde{H}_{\mathrm{eff}}= \Delta\sum_{\sigma} \left( \gamma_{K,+,\sigma}^{\dagger}\gamma_{K,+,\sigma}-\gamma_{K,-,\sigma}^{\dagger}\gamma_{K,-,\sigma}-\gamma_{K^{'},+,\sigma}^{\dagger}\gamma_{K^{'},+,\sigma}+\gamma_{K^{'},-,\sigma}^{\dagger}\gamma_{K^{'},-,\sigma} \right)+\omega_{0}a^{\dagger}a+g\sum_{e_{Y},\sigma} \left( \gamma_{K,e_{Y},\sigma}^{\dagger}\gamma_{K^{'},e_{Y},\sigma}+h.c. \right)\left( a+a^{\dagger} \right)+\frac{g^{2}}{\omega_{0}}{(\sum_{e_{Y},\sigma} \gamma_{K,e_{Y},\sigma}^{\dagger}\gamma_{K^{'},e_{Y},\sigma}+h.c.)}^{2}$$

*(61)*

where the tunable parameter $\Delta$ reflects the hBN potential energy, and $g$ is the electron-phonon coupling coefficient. The phonon frequency $\omega_{0}$ is set to 150 meV. Since different momentum sectors ($\bar{\boldsymbol{k}}$) interacts indirectly only through the coupling to the same phonons, this two-site four-orbital model provides a qualitative approximation for the chiral-limit TBG/hBN systems with the finite-size effect renormalizing the coupling strength. In the ED simulation, we further considered the electronic interaction in the form of ${g^{2}}/{\omega_{0}}$ in Eq. (61), which has been ignored in variational non-Gaussian simulations in Sec. IX-XI. Such a repulsion term compensates the phonon-induced effective attraction at the $\Delta=0$ (TBG) limit, leading to a gapless band structure consistent with experiments.

With this toy-model Hamiltonian, we simulate the spectral function precisely using Eq. (25)

$A\left( \omega\right)=\mathrm{Im}\left\{ \left\langle G | \gamma^{\dagger}\frac{1}{\omega-\tilde{H}_{\mathrm{eff}}+E_{G}-i\Gamma}\gamma| G \right\rangle\right\}+\mathrm{Im}\left\{ \left\langle G | \gamma\frac{1}{\omega+\tilde{H}_{\mathrm{eff}}-E_{G}-i\Gamma}\gamma^{\dagger} | G \right\rangle\right\}$ *(62)*

(Note that we do not define momentum in such a two-site four-orbital system. So the spectral function $A\left( k,\omega\right)$ is reduced to $A\left( \omega\right)$.) The simulated spectral function is presented in Fig. 4 of the main text for an elevated coupling strength $g=$100 meV for best contrast, while Fig. S13(a) also presents the results with $g=$50 meV, where the replica intensity reflects the correct ratio corresponding to the iTO phonon derived from Sec. IX (with the Poisson factor ~ 0.11). This result reflects the mechanism discussed in Sec. X. Due to the non-commuting nature of the hBN potential (controlled by $\Delta$ ) and the EPC (controlled by $g$), they compete in determining the polaronic dressing in the ground-state wavefunction. This effect is parametrized as $\lambda$ in the variational ansatz discussed in Sec. X. While the ED solution does not provide an intuitive parameter, the phonon-induced spectral replica depicts the effective polaronic dressing. As shown in Fig. S13(a), the polaron replica is suppressed when a finite hBN potential $\Delta$ is introduced. With the increase of $\Delta$, the replica rapidly fades away and the spectral returns to the non-interacting $\delta$-functions (separated by the hBN potential).

To further demonstrate the importance of the band inversion induced by hBN, we consider an artificial model where the two bands in both valleys shift in parallel.

$$\tilde{H}_{\mathrm{eff}}= \Delta\sum_{\sigma} \left( \gamma_{K,+,\sigma}^{\dagger}\gamma_{K,+,\sigma}-\gamma_{K,-,\sigma}^{\dagger}\gamma_{K,-,\sigma}+\gamma_{K^{'},+,\sigma}^{\dagger}\gamma_{K^{'},+,\sigma}-\gamma_{K^{'},-,\sigma}^{\dagger}\gamma_{K^{'},-,\sigma} \right)+\omega_{0}a^{\dagger}a+g\sum_{e_{Y},\sigma} \left( \gamma_{K,e_{Y},\sigma}^{\dagger}\gamma_{K^{'},e_{Y},\sigma}+h.c. \right)\left( a+a^{\dagger} \right)+\frac{g^{2}}{\omega_{0}}{(\sum_{e_{Y},\sigma} \gamma_{K,e_{Y},\sigma}^{\dagger}\gamma_{K^{'},e_{Y},\sigma}+h.c.)}^{2}$$

*(63)*

Using this model, we further simulate the spectral distribution and its dependence on $\Delta$. As shown in Fig. S13(b), the presence of $\Delta$ does not suppress the replica while opening the gap. Though this small-cluster toy model is not delicate enough to quantitatively describe the physics in TBG/hBN, it provides additional intuitions and verifications for the band inversion mechanism discussed in Sec. X. As a side note, we can also deduce the bandwidth effect from this ED simulation, by recognizing the fact that momentum contributes to an additional band inversion in Eq. (40). Intuitively, it can be interpreted as allowed particle-hole states for electron-phonon scattering is reduced when the corresponding bands leave their nesting condition.

Using this two-site toy model, we can further analyse the impact of electronic Coulomb interactions qualitatively, which cannot be directly evaluated in Sec. IX-XI due to their many-body nature. Specifically, we consider the Hubbard-like interactions, including the on-site repulsion denoted as $U$ and the inter-orbital Coulomb repulsion denoted as $U^{'}$, with ${U= U}^{'}$ in the chiral basis. These two interactions are found dominant over others (e.g. the Hund’s exchange and nonlocal interactions)^35^. That leads to the additional terms in the Hamiltonian

$\tilde{H}_{\mathrm{int}}= \frac{U}{2}\sum_{\begin{aligned} \eta,\eta^{'} \\ e_{Y} \end{aligned}} \gamma_{\eta-q,e_{Y},\uparrow}^{\dagger}\gamma_{\eta,e_{Y},\uparrow}\gamma_{\eta^{'}+q,e_{Y},\downarrow}^{\dagger}\gamma_{\eta^{'},e_{Y},\downarrow}+\frac{U}{2}\sum_{\begin{aligned} \eta,\eta^{'},q \\ \sigma,\sigma^{'} \end{aligned}} \gamma_{\eta-q,+,\sigma}^{\dagger}\gamma_{\eta,+,\sigma}\gamma_{\eta^{'}+q,-,\sigma^{'}}^{\dagger}\gamma_{\eta^{'},-,\sigma^{'}}$ *(64)*

Adding the interaction part into the Hamiltonian $\tilde{H}_{\mathrm{eff}}$, the simulated PES spectrum is depicted in Fig. S14. We tune the local Coulomb interaction $U$ from 0 to 60 meV under room temperature (300 K). As $U$ increases, a Mott gap opens up, separating singly and doubly occupied states. Due to the flat-band assumption, this Mott gap size is set by $U$; however, the realistic gap should be reduced by the single-particle bandwidth. Since the phonon energy is much larger than the Coulomb interaction, such a Mott gap can be distinguished from the polaronic replica. Notably, the inclusion of this Coulomb interaction $\tilde{H}_{\mathrm{int}}$ does not affect the spectral weights of these replicas. Mathematically, this independence arises from the absolute locality of the on-site Coulomb interaction, which is identical in valley basis and commutes with the inter-valley EPCs.


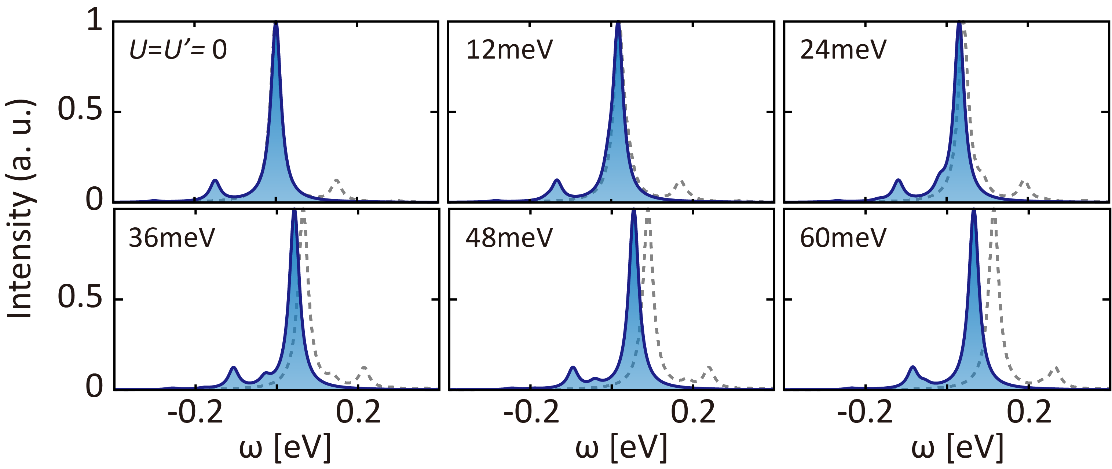


**Fig. S14 | Influence of electronic Coulomb interactions.** Electron removel spectral function obtaind for the two-site TBG model using ED. The local Coulomb interaction (intra-band Hubbard $U$ and inter-band $U^{'}$) strength is tuned from 0 to 60 meV, while the EPC is $g=50 meV,$and phonon energy $\omega_{0}=150 \mathrm{meV}$. The simulation is conducted at room temperature $T=$300 K and a lifetime broadening$\Gamma=15 \mathrm{me}$V is added.

**XIII Strong correlation between the phonon replicas of the flat bands and superconductivity in MATBG**

In this section, we explain how a strong correlation between the phonon replicas of the flat band and the superconductivity in MATBG can be naturally established, from both experimental results and theoretical analysis/calculations.

With the experimental results accumulated so far, we can summarize a table illustrating the correlation between the flat band’s phonon replicas and the superconductivity, as shown in Fig. S15. It’s evident that for ALL non-superconducting samples – either hBN aligned MATBG or TBGs with twisted angle deviating from the magic angle – no flat band replicas were observed. The ONLY case where flat band replicas were observed was in the superconducting, hBN non-aligned samples. These experimental outcomes naturally indicate a strong correlation/coincidence between the presence of superconductivity in MATBG and the presence of phonon replicas.

.
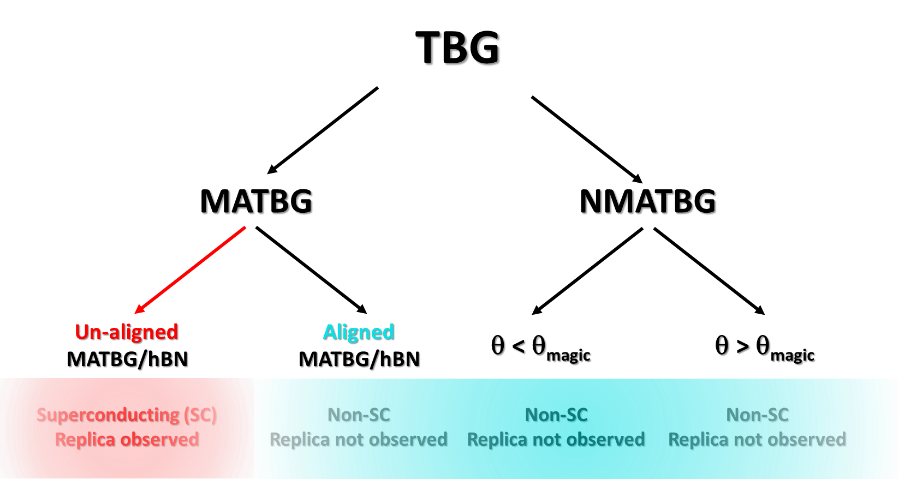


**Fig. S15|** **A brief summary of current experimental results.** Acronyms: TBG: twisted bi-layer graphene; MATBG: Magic angle twisted bi-layer graphene; NMATBG: Non-magic angle twisted bi-layer graphene. SC: Superconducting; Non-SC: Non-superconducting.

On the theory side, our analysis and simulations, presented in the Method session and the SI Section VI-XII, not only demonstrate the emergence of phonon replicas in superconducting samples due to intervalley electron-phonon coupling, but also explain the absence of replicas in non-superconducting samples – either due to hBN alignment with MATBG or deviations in the twist angle from the magic angle. The interplay between electron-phonon coupling and Coulomb interaction in this system is also discussed.

Moreover, in our separated theory work^27^, we further develop an analytical theory to describe the interaction between electrons and the inter-valley iTO phonons and study its influence on superconductivity in MATBG based on symmetry analysis and linearized gap equations. From the linearized gap equations, we find that the highest *T_c_* pairing induced by this phonon is a singlet gapped s-wave inter-Chern-band order parameter, followed closely by a gapless nematic d-wave intra-Chern-band order parameter. The latter state is highly compatible with several existing experiments that suggest nodal superconductivity in magic-angle twisted bilayer and trilayer graphene^5,36-39^, which further supports the likely importance of intervalley iTO phonons to superconductivity in both materials.

In summary, the strong correlation between the phonon replicas and the superconductivity in MATBG is not only evident from the experimental results (Fig. S15), but also supported by the theory and simulations, which include strong electron-phonon coupling, as presented in the Method and SI Sections VI-XII. These findings not only explain all the experimental observations in the current work, but also support a superconducting ground state with s- or d-wave order parameters^27^.

**References:**

1. Sharpe, A. L. et al. Emergent ferromagnetism near three-quarters filling in twisted bilayer graphene. *Science* **365**(6453), 605-608 (2019).
2. Serlin, M. et al. Intrinsic quantized anomalous Hall effect in a moiré heterostructure. *Science* **367**, 900-903 (2020).
3. Shi, J. et al. Moiré commensurability and the quantum anomalous hall effect in twisted bilayer graphene on hexagonal boron nitride. *Phys. Rev. B*, **103**, 075122, (2021).
4. Lin, X. et al. Misalignment instability in magic-angle twisted bilayer graphene on hexagonal boron nitride. *2D Mater.*, **8**, 025025, (2021).
5. Oh, M. et al. Evidence for unconventional superconductivity in twisted bilayer graphene. *Nature* **600**, 240-245 (2021).
6. Cao, Y. et al. Unconventional superconductivity in magic-angle graphene superlattices. *Nature* **556**, 43–50 (2018).
7. Lu, X. et al. Superconductors, orbital magnets and correlated states in magic-angle bilayer graphene. Nature **574**, 653–657 (2019).
8. Yankowitz, M. et al. Tuning superconductivity in twisted bilayer graphene. *Science*, **363**(6431), 1059-1064, (2019).
9. He, Y. et al. Superconducting fluctuations in overdoped Bi_2_Sr_2_CaCu_2_O_8+ δ_. *Phys. Rev. X*, **11**, 031068 (2021).
10. Utama, M. I. B. et al. Visualization of the flat electronic band in twisted bilayer graphene near the magic angle twist. *Nat. Phys.* **17**, 184-188, (2021).
11. Zhang, Y., et al. Giant phonon-induced conductance in scanning tunnelling spectroscopy of gate-tunable graphene. *Nat. Phys.* **4**, 627-630 (2008).
12. Zhao, Y. et al. Creating and probing electron whispering-gallery modes in graphene. *Science* **348**, 672–675 (2015).
13. Lee, J. et al. Imaging electrostatically confined Dirac fermions in graphene quantum dots. *Nat. Phys.* **12**, 1032–1036 (2016).
14. Jiang, Y. et al. Tuning a circular p–n junction in graphene from quantum confinement to optical guiding. *Nat. Nanotechnol.* **12**, 1045–1049 (2017).
15. Ge, Z. et al. Giant orbital magnetic moments and paramagnetic shift in artificial relativistic atoms and molecules. *Nat. Nanotechnol.* **18**, 250–256 (2023).
16. Wong, D. et al. Cascade of electronic transitions in magic-angle twisted bilayer graphene. *Nature* **582**, 198–202 (2020).
17. Koshino, M. et al. Maximally localized wannier orbitals and the extended hubbard model for twisted bilayer graphene. *Phys. Rev. X*, **8**(3), 031087, (2018).
18. Moon, P. et al. Optical absorption in twisted bilayer graphene. *Phys. Rev. B*, 87(20), 205404, (2013).
19. Miao, W. et al. Truncated Atomic Plane Wave Method for the Subband Structure Calculations of Moiré Systems. *Phys. Rev. B*, **107**, 125112 (2023).
20. Choi, Y. W. et al. Dichotomy of electron-phonon coupling in graphene moiré flat bands. *Phys. Rev. Lett.*, **127**(16), 167001, (2021).
21. Choi, Y. W. et al. Strong electron-phonon coupling, electron-hole asymmetry, and nonadiabaticity in magic-angle twisted bilayer graphene. *Phys. Rev. B*, **98**(24), 241412, (2018).
22. Liu, X. et al. Moiré Phonons in Magic-Angle Twisted Bilayer Graphene. *Nano letters*, **22**(19), 7791-7797, (2022).
23. Angeli, M. et al. Valley Jahn-Teller effect in twisted bilayer graphene. *Phys. Rev. X*, 9(4), 041010, (2019).
24. Angeli, M. et al. Jahn–Teller coupling to moiré phonons in the continuum model formalism for small-angle twisted bilayer graphene. *Eur. Phys. J. Plus*, **135**(8), 1–17 (2020).
25. Hao, S. et al. Moiré optical phonons dancing with heavy electrons in magic-angle twisted bilayer graphene. *arXiv preprint* arXiv:2402.11824 (2024).
26. Esterlis, I. et al. Breakdown of the Migdal-Eliashberg theory: A determinant quantum Monte Carlo study. *Phys. Rev. B* 97, 140501(R) (2018).
27. Liu, C. et al. Electron-K Phonon Interaction In Twisted Bilayer Graphene, *arXiv preprint* arXiv:2303.15551 (2023).
28. I. G. Lang et al. *Sov. Phys. JETP* **16**, 1301, (1962).
29. Shi, J. et al. Moiré commensurability and the quantum anomalous Hall effect in twisted bilayer graphene on hexagonal boron nitride. *Phys. Rev. B*, **103**, 075122 (2021).
30. Hunt, B. et al. Massive Dirac fermions and Hofstadter butterfly in a van der Waals heterostructure. *Science*, **340**, 1427-1430 (2013).
31. Shi, T. et al. Variational study of fermionic and bosonic systems with non-Gaussian states: Theory and applications. *Annals of Physics*, **390**, 245-302 (2018).
32. Wang, Y. et al. Fluctuating nature of light-enhanced d-wave superconductivity: a time-dependent variational non-gaussian exact diagonalization study. *Phys. Rev. X*, **11**, 041028 (2021).
33. Mishchenko, A.S. et al. Electron-phonon coupling and a polaron in the t− J model: from the weak to the strong coupling regime. *Phys. Rev. Lett.*, **93**, 036402 (2004).
34. Wang, Y. et al. Zero-temperature phases of the two-dimensional Hubbard-Holstein model: A non-Gaussian exact diagonalization study. *Phys. Rev. Res.*, **2**, 043258 (2020).
35. Song, Z. D., & Bernevig, B. A. Magic-angle twisted bilayer graphene as a topological heavy fermion problem. *Phys. Rev. Lett.*, **129**(4), 047601 (2022).
36. Kim, H. et al. Evidence for unconventional superconductivity in twisted trilayer graphene. *Nature* **606**, 494–500 (2022).
37. Di Battista, G. et al. Revealing the thermal properties of superconducting magic-angle twisted bilayer graphene. *Nano Lett.* **22**(16), 6465-6470 (2022).
38. Tanaka, M. et al. Kinetic Inductance, Quantum Geometry, and Superconductivity in Magic-Angle Twisted Bilayer Graphene. *arXiv preprint arXiv:2406.13740* (2024).
39. Banerjee, A. et al. Superfluid stiffness of twisted multilayer graphene superconductors. *arXiv preprint arXiv:2406.13742* (2024).
